# Lutein Exerts Antioxidant and Anti-Inflammatory Effects and Influences Iron Utilization of BV-2 Microglia

**DOI:** 10.3390/antiox10030363

**Published:** 2021-02-27

**Authors:** Ramóna Pap, Edina Pandur, Gergely Jánosa, Katalin Sipos, Attila Agócs, József Deli

**Affiliations:** 1 Department of Pharmaceutical Biology, Faculty of Pharmacy, University of Pécs, Rókus u. 2., H-7624 Pécs, Hungary; pap.ramona@pte.hu (R.P.); edina.pandur@aok.pte.hu (E.P.); gergely.janosa@gytk.pte.hu (G.J.); katalin.sipos@aok.pte.hu (K.S.); 2 Department of Biochemistry and Medical Chemistry, Medical School, University of Pécs, Szigeti út 12., H-7624 Pécs, Hungary; attila.agocs@aok.pte.hu; 3 Department of Pharmacognosy, Faculty of Pharmacy, University of Pécs, Rókus u. 2., H-7624 Pécs, Hungary

**Keywords:** microglia, lutein, iron, hydrogen-peroxide, cytokines

## Abstract

Lutein is a tetraterpene carotenoid, which has been reported as an important antioxidant and it is widely used as a supplement. Oxidative stress participates in many human diseases, including different types of neurodegenerative disorders. Microglia, the primary immune effector cells in the central nervous system, are implicated in these disorders by producing harmful substances such as reactive oxygen species (ROS). The protective mechanisms which scavenge ROS include enzymes and antioxidant substances. The protective effects of different carotenoids against oxidative stress have been described previously. Our study focuses on the effects of lutein on antioxidant enzymes, cytokines and iron metabolism under stress conditions in BV-2 microglia. We performed cell culture experiments: BV-2 cells were treated with lutein and/or with H_2_O_2_; the latter was used for inducing oxidative stress in microglial cells. Real-time PCR was performed for gene expression analyses of antioxidant enzymes, and ELISA was used for the detection of pro- and anti-inflammatory cytokines. Our results show that the application of lutein suppressed the H_2_O_2_-induced ROS (10′: 7.5 ng + 10 µM H_2_O_2_, *p* = 0.0002; 10 ng/µL + 10 µM H_2_O_2_, *p* = 0.0007), influenced iron utilization and changed the anti-inflammatory and pro-inflammatory cytokine secretions in BV-2 cells. Lutein increased the IL-10 secretions compared to control (24 h: 7.5 ng/µL *p* = 0.0274; 10 ng/µL *p* = 0.0008) and to 10 µM H_2_O_2_-treated cells (24 h: 7.5 ng/µL + H_2_O_2_, *p* = 0.0003; 10 ng/µL + H_2_O_2_, *p* = 0.0003), while it decreased the TNFα secretions compared to H_2_O_2_ treated cells (24 h: 7.5 ng/µL + H_2_O_2_, *p* < 0.0001; 10 ng/µL + H_2_O_2_, *p* < 0.0001). These results contribute to understanding the effects of lutein, which may help in preventing or suppressing ROS-mediated microglia activation, which is related to neuronal degeneration in oxidative stress scenario.

## 1. Introduction

Carotenoids are representatives of naturally occurring tetraterpenes and are present in all plant organs; in addition, they can be found in human and animal organisms. According to the latest reports, about 850 naturally occurring carotenoids can be synthetized by several microbes, fungi, different algae and higher plants [1]. Lutein is a well-studied xanthophyll carotenoid, which is widely used as dietary supplement in protection against age-related degenerative eye disorders such as macular degeneration and cataracts [2]. Furthermore, lutein has been reported with antioxidant and anti-inflammatory properties [3,4].

Several antioxidants have been used to prevent and relieve oxidative damage associated with diseases [5]. Oxidative stress is one of the components involved in many human diseases, including different types of cancers [6]. The protective mechanisms which scavenge reactive oxygen species (ROS) include enzymes and antioxidant substances. A large number of scientific papers deal with the protective effects of different carotenoids against oxidative stress [7,8].

Microglia, the primary immune effector cells of the central nervous system (CNS), have been associated with major function in maintaining brain homeostasis and regulating neuronal surveillance. The ROS produced by either microglia or in the surrounding environment are able to influence neuronal signalling and also to modify the microglial activities [9,10]. Interleukin-10 (IL-10) can be released by microglia as an important modulator cytokine and is capable of controlling the anti-inflammatory effects and the neuroprotective functions through which microglia can modulate the homeostasis of the CNS [11,12]. Tumor necrosis factor α (TNFα) participates in the management of the immune response and in the protection from infections and has a crucial role in several autoimmune and neurodegenerative diseases. In the CNS, the microglia activation attendant overproduction of TNFα has been implicated in neuronal death [13,14].

Mitochondrion is the pivotal centre of intracellular iron metabolism; moreover, it has a major role in the regulation of redox mechanisms [15]. Antioxidants are supported by antioxidant enzymes including superoxide dismutase and catalase, among others, which exert synergistic actions in removing free radicals. The mitochondrial superoxide dismutase 2 (SOD2) is known to play a crucial role in stress induced diseases, also used as a biomarker in some human disorders, and is the only known enzyme which is essential physiologically for aerobic survival [16,17]. Catalase, as a member of the essential antioxidant enzymes, is able to alleviate oxidative stress via decomposing cellular hydrogen peroxide to oxygen and water [18]. The heat shock protein family member heme oxygenase-1 (HO-1) is a crucial component of the cellular stress response and is known to have antioxidant, anti-inflammatory and protective roles against tissue injury. In addition, HO-1 is required for the degradation of heme [19,20]. 

Iron is a vitally important element of brain function regulations inter alia with the role in oxidative metabolisms, neurodegenerative disorders and inflammatory responses via microglia [21]. The iron–sulfur cluster (Fe-S) synthesis and the major part of the heme synthesis enzymes are located in the mitochondria with the heme and Fe-S cluster-containing proteins [15]. The terminal step of heme biosynthesis is catalysed by Ferrochelatase (FECH), which enhances the insertion of the imported iron into protoporphyrin IX to produce heme. The iron transporter mitoferrin 2 is located on the inner membrane of mitochondria and is liable for the mitochondrial import of Fe^2+^ from the intermembrane space to the matrix [22]. Inside mitochondria, the iron is incorporated into heme and Fe-S clusters. Heme is a conjugate complex which consists of a central iron and porphyrin IX produced in mitochondria. It is also an important signalling molecule in physiological processes [23,24]. Fe-S cluster proteins serve as cofactors of a large number of enzymes performing essential biological functions. For their assembly, a mitochondrial cysteine desulfurase (NFS1) provides sulfur donor in the form of a persulfide [25]. The mitochondrion also possesses iron storage capacity by mitochondrial ferritin (FTMT), which provides protection against oxidative damage [26]. In this study, we examined the antioxidant and anti-inflammatory effects of lutein in the presence of H_2_O_2_ in BV-2 microglia and its possible role in the regulation of iron metabolism. Based on our results we suppose that lutein acts against ROS production and decreases inflammation by reducing TNFα secretion and increasing IL-10. Lutein influenced mitochondrial iron homeostatis by increasing mitochondrial iron storage protein levels and by modulating heme and iron sulfur cluster syntheses. 

## 2. Materials and Methods

### 2.1. Isolation of Lutein

The Marigold extract (INEXA, Industria Extractora C.A., Quito, Ecuador) was dissolved in diethyl ether and saponified overnight with 30% KOH in methanol. The etherial solution was washed with water five times, then dried and evaporated. The crude saponified extract was crystallized from hexane/toluene to deliver lutein with 98% purity. According to HPLC, on a C30 column lutein contained 6% zeaxanthin. Dimethyl sulfoxide (DMSO, Sigma-Aldrich Kft., Budapest, Hungary) was used as a carrier solvent for lutein, which was diluted in the concentration of 1 mg/mL. The diluted stock was sterile filtered and stored at −20 °C, protected from light.

### 2.2. Cell Culture and Treatments

BV-2 murine microglial cells (a generous gift from Prof. László Tretter and his research group) were maintained in Dulbecco’s Modified Eagle Medium (DMEM; Lonza Ltd., Basel, Switzerland) supplemented with 10% fetal bovine serum (FBS, Biowest SAS, Nuaillé, France) and 1% penicillin/streptomycin (Lonza Ltd., Basel, Switzerland). The cells were cultured on poly-L-ornithine- (Sigma-Aldrich Kft., Budapest, Hungary) coated dishes (Sarstedt Kft., Budapest, Hungary). For the experiments, BV-2 cells were seeded onto 6-well plates or onto 60 × 15 mm dishes in antibiotic free complete growth medium, and were grown for 24 h before the treatments in all experiments. In the preliminary experiments, the cells were treated or pre-treated for 24 h with lutein at the concentration of 2.5, 5, 7.5 and 10 ng/µL; in the other experiments, the cells were treated with 5, 6, 7, 8, 9, 10, 15, 25, 50, 75, 100, 200, 300, 400 and 500 µM of hydrogen peroxide (H_2_O_2_) (Sigma-Aldrich Kft., Budapest, Hungary). In the further experiments the cells were treated with 7.5 and 10 ng/µL of lutein and/or with 10 µM of H_2_O_2_. Untreated cells were used as absolute controls. DMSO treated cells were used as controls of the lutein treated cells. We used the following abbreviations for the treatments: C marks the appropriate untreated absolute control at 6, 24 and 48 h. DMSO controls: D7 means the same volume of DMSO as 7.5 ng/µL of lutein was added. D10 means the same amount of DMSO as 10 ng/µL of lutein was used for the treatments. The cells which were treated with lutein and H_2_O_2_ together received the treatments at the same time: the lutein first, which was followed by H_2_O_2_. Each experiment was repeated at least three times. All experiments were carried out in a humidified atmosphere containing 5% CO_2_ at 37 °C.

### 2.3. Cell Viability Assay

BV-2 cells were plated onto 96-well plates using 5 × 10^3^ cells/well. Cells were treated with lutein or with H_2_O_2_ for 2, 6, 24 and 48 h. The following concentrations were used for the measurements in the H_2_O_2_ viability tests: 5, 6, 7, 8, 9, 10, 15, 25, 50, 75, 100, 200, 300, 400 and 500 µM of H_2_O_2_. The following concentrations were used for the measurements in the lutein viability assays: 2.5, 5, 7.5 and 10 ng/µL of lutein. The viability of the BV-2 cells was measured using Cell Counting Kit-8 (CCK-8) cell viability assay (Sigma-Aldrich Kft., Budapest, Hungary) after the treatments. DMSO control cells were treated with an amount equal to lutein treatment and served as controls of each appropriate lutein-treated cells, while the effect of DMSO on cell viability was determined by using untreated cells as controls. We used the following abbreviations for treatments: Control—absolute control; DMSO controls: D7—DMSO equivalent to 7.5 ng/µL of lutein; D10—DMSO equivalent to 10 ng/µL of lutein; D5—DMSO equivalent to 5 ng/µL of lutein; D2—DMSO equivalent to 2.5 ng/µL of lutein. After each treatment, 10 µL of WST-8 reagent was added to each well, then the plates were incubated for 1 h at 37 °C and 5 % CO_2_. After incubation, the absorbance of the samples was measured at 450 nm using MultiSkan GO microplate spectrophotometer (Thermo Fisher Scientific Inc., Waltham, MA, USA). Viability was expressed as percentile of the total cell number of the appropriate control cells.

### 2.4. Detection of the Reactive Oxygen Species

Cellular ROS Assay Kit (Deep Red) (Abcam, Cambridge, UK) was used for oxidative stress detection. According to the results of the cell viability measurements, 5 and 10 µM of H_2_O_2_ was used to generate ROS in vitro in the BV-2 cells. A total of 5 × 10^3^ cells/well were plated for the measurements. The cells were exposed to treatments for 24 h. To check the possible effect of lutein for the ROS generation, freshly added lutein and 24 h of lutein pre-treatment was used for the cells before the H_2_O_2_ was added. The freshly added lutein was followed by adding H_2_O_2_ immediately. The assay used an ROS sensor to quantify ROS in live cells. The assay was performed in a 96-well microtiter plate. The experiments were carried out according to the manufacturers’ instructions. Briefly, after 24 h of resting state, the control, the lutein-treated and lutein-pre-treated cells were measured or treated with different concentrations of H_2_O_2_ to produce ROS and incubated for 10, 20 and 30 min at 37 °C and 5% CO_2_. After incubation, the cells were stained and incubated for 30 min at 37 °C and 5% CO_2_ with ROS deep red dye solution (100 µL/well). The absorbance was measured using EnSpire Multimode microplate reader (PerkinElmer, Rodgau, Germany) at an excitation wavelength of 650 nm and an emission wavelength of 675 nm with bottom read mode. The ROS changes were determined as a percentage of control.

### 2.5. Real-Time PCR

BV-2 cells were treated the same way as described above, in 6-well culture dishes (3 × 10^5^ cells/well). After the treatments, BV-2 cells were washed with PBS and then were collected from the 6-well plates after trypsinization. Quick-RNA™ MiniPrep Kit (Zymo Research, Irvine, CA, USA) was used for total RNA isolation from each sample according to the manufacturers’ instructions. Complementary DNA was synthesized from 200 ng of total RNA using High Capacity cDNA Reverse Transcription Kit (Applied Biosystems, Thermo Fisher Scientific Inc., Waltham, MA, USA) according to the manufacturers’ protocol. Determination of gene expressions was performed in a CFX96 Real-Time PCR System (Bio-Rad Inc., Hercules, CA, USA) using iTaq™ Universal SYBR^®^ Green Supermix (Bio-Rad Inc., Hercules, CA, USA) in a 20 µL of total reaction volume. Melting curves were generated after each quantitative PCR run to ensure that a single specific product was amplified. Relative quantification was calculated by the 2^-ΔΔCq^ (Livak) method using the Bio-Rad CFX Maestro 1.1 software (Bio-Rad Inc., Hercules, CA, USA). The expression level of the gene of interest was compared with the level of internal control β-actin in each sample. These relative expression rates were then compared between the treated and the untreated samples. The relative expression of the controls was regarded as 1. The mRNA expression of the treated cells was compared to the appropriate controls. The primer sequences used in this study are described in Table 1.

### 2.6. Enzyme-Linked Immunosorbent Assay (ELISA) Measurements

Microglial cells were cultured as described above. Culture media were collected after each treatment of the cells and stored at −80 °C until the measurements. The anti-inflammatory, antioxidant IL-10 and the pro-inflammatory, pro-oxidant TNFα cytokine concentrations of the culture media were detected using mouse IL-10 and mouse TNFα ELISA Kits (Invitrogen, Thermo Fisher Scientific Inc., Waltham, MA, USA) according to the assay procedure instructions of the manufacturer. The absorbance was measured by using a MultiSkan GO Microplate Spectrophotometer (Thermo Fisher Scientific Inc., Waltham, MA, USA) at 450 nm. The emission intensity of the signal is directly related to the concentration of target present in the original samples. The concentrations of cytokines were expressed as pg/mL.

### 2.7. Measurements of Total Iron

A ferrozine-based colorimetric assay was used to perform the determination of the iron concentration of the BV-2 cells, which is described by Riemer et al. [27]. Briefly, the BV-2 cells were collected and lysed with 50 mM NaOH at room temperature for 2 h. After the incubation, the samples were mixed with iron releasing reagent (1.4 M HCl, 4.5% (wt/vol) KMnO_(4)_ in dH_2_O) and were incubated at 60 °C for 2 h. Thereafter an iron detection reagent (6.5 mM ferrozine, 6.5 mM neocuproine, 1 M ascorbic acid and 2.5 M ammonium acetate) was added to each tube and incubated at room temperature for 30 min. The absorbance was measured using a MultiSkan GO Spectrophotometer at 550 nm (Thermo Fisher Scientific Inc., Waltham, MA, USA). The concentration was determined by a standard curve of FeCl_3_ (0–300 µM) treated the same way as the samples. A DC Protein Assay Kit was used to measure the protein concentration of each sample (Bio-Rad Inc., Hercules, CA, USA). The iron content was normalized against the protein content and was expressed as µM iron/mg protein.

### 2.8. Measurements of Heme Concentration

The heme concentration was determined with Heme assay kit (Sigma-Aldrich Kft., Hungary), which is an aqueous alkaline solution method-based assay. The assay procedure was performed according to the manufacturers’ instructions. Briefly, after the treatments, the BV-2 cells were collected and lysed with 50 µL of water. An amount of 50 µL of water was used as a blank. An amount of 50 µL of water and 50 µL of Heme Calibrator were measured into wells of a 96-well plate, then 200 µL of water into each of the blank and Heme Calibrator reaction wells. Next, 50 µL of samples were added into wells, followed by using 200 µL of Heme Reagent added to each sample well. The plate was incubated for 5 min at room temperature. All samples and standards were run in triplicate in four independent experiments. The diluted Heme Calibrator is equivalent to 62.5 M heme. The heme is converted into a uniformly colored form, producing a colorimetric result, which can be measured at 400 nm and is directly proportional to the heme concentration in the sample. The absorbance was measured using a MultiSkan GO Microplate Spectrophotometer (Thermo Fisher Scientific Inc., Waltham, MA, USA). The concentration of heme was expressed as µM.

### 2.9. Western Blotting

BV-2 cells were seeded onto 60 × 15 mm dishes (6 × 10^5^ cells/well) and were treated after a 24 h incubation period. To provide protein extraction, 200 µL of lysis buffer (50 mM Tris-HCl, pH 7.4, 150 mM NaCl, 0.5% Triton-X 100) was used with a complete mini protease inhibitor cocktail (Roche Ltd., Basel, Switzerland) after collection of the cells. Protein contents of the solutions were measured with a DC Protein Assay Kit (Bio-Rad Inc., Hercules, CA, USA). The same amount of protein (30 µg) from each sample was loaded onto 12% polyacrylamide gels. A Bio-Rad Mini Protean Tetra Cell (Bio-Rad Inc., Hercules, CA, USA) was used for electrophoresis. After the electrophoresis, the protein content of the gels were transferred by electro blotting to nitrocellulose membranes (Pall AG, Basel, Switzerland). The membranes were blocked with 5% non-fat dry milk (Bio-Rad Inc., Hercules, CA, USA) in TBST (Tris-buffer saline, 0.1% Tween-20) for 1 h at room temperature. After the blocking step, the membranes were probed with the following polyclonal rabbit antibodies for 1 h at room temperature according to the manufacturers’ protocols: anti-SOD2 IgG (1:1000, GeneTex Inc., Irvine, CA, USA), anti-NFS1 IgG (1:1000, Novus Biologicals, Bio-Techne, Minneapolis, MN, USA) anti-FTMT IgG (1:500, Termo Fisher Scientific Inc., Waltham, NA, USA) and anti-Fc IgG (1:1000, Novus Biologicals, Bio-Techne, Minneapolis, MN, USA), and overnight at 4 °C in the case of anti-HO-1 IgG (1:1000, Cell Signaling Technology Europe, Leiden, The Netherlands). Anti-GAPDH (1:3000, Merck KGaA, Darmstadt, Germany) was used as loading control in all Western blot experiments. Goat anti-rabbit HRP-conjugate was used as secondary antibody (1:2000, Cell Signaling Technology Europe, Leiden, The Netherlands). We applied traditional colorimetric detection Fuji Medical X-ray Film (Fujifilm Corporation, Tokyo, Japan). Protein detection was carried out with WesternBright ECL chemiluminescent substrate (Advansta Inc., San Jose, CA, USA). Optical densities of Western blots were determined using ImageJ software [28] and were expressed as percentage of target protein/GAPDH abundance.

### 2.10. Statistical Analysis

The presented data are representative of at least three independent experiments. For all data, n corresponds to the number of independent experiments. Real-time PCR, ELISA assays, total iron measurements and heme measurements were carried out in triplicate in each independent experiment. Cell viability assays and ROS assays were carried out in quadruplicate in each independent experiment. Statistical analysis was performed using SPSS software (IBM Corporation, Armonk, NY, USA). Statistical significance was determined using ANOVA analyses with Tukey HSD post-hoc tests to compare treated groups (6, 24 and 48 h) to their appropriate control group (6, 24 and 48 h) and to calculate the significant difference between treated groups (e.g., lutein vs. lutein with H_2_O_2_). Data are shown as mean values with error bars corresponding to the standard deviation (SD). Statistical significance was set at *p* value < 0.05.

## 3. Results

### 3.1. Lutein Suppresses the H_2_O_2_-Induced ROS in BV-2 Cells

ROS are the pivotal molecules responsible for the harmful effects of oxidative stress. As an exogenous source, hydrogen peroxide (H_2_O_2_) was used to induce ROS in BV-2 cells. First, we determined the working concentrations of lutein and H_2_O_2_ by applying concentration and time-dependent preliminary viability experiments. The BV-2 cells treated with 7.5 and 10 ng/µL of lutein showed an increased viability at 6, 24 and 48 h (Figure S1) compared to the appropriate control cells and H_2_O_2_ treated cells (Table S1). Based on the viability results of H_2_O_2_ treated cells (Figure S2), 5 and 10 µM of H_2_O_2_ were chosen for inducing oxidative stress for the ROS measurements in BV-2 cells.

**Figure 1:**
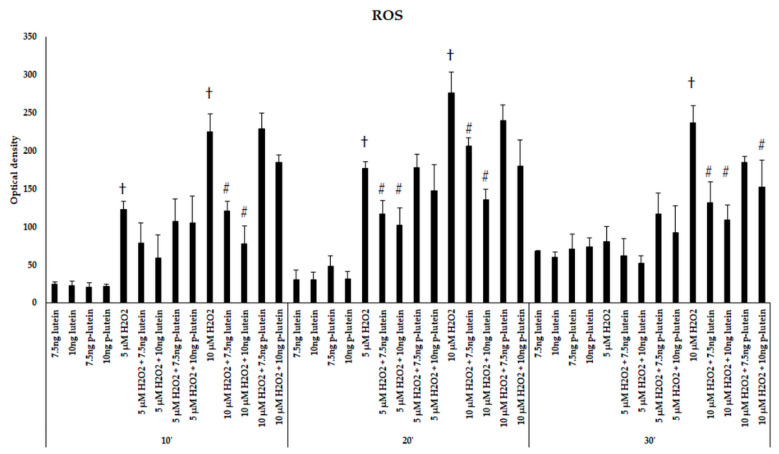
Reactive oxygen species detection after lutein and H_2_O_2_ treatment in BV-2 cells. The columns represent the mean values and error bars indicate the standard deviation (SD) of three independent experiments (*n* = 3), each performed in quadruplicate, and are presented relative to own control cells. † indicates statistical significance of H_2_O_2_ treated cells compared to controls, # marks statistical significance of combined (lutein with H_2_O_2_) treatments compared to H_2_O_2_ treatments; the level of significance was *p* < 0.05. Abbreviations: p-lutein is 24 h of lutein pre-treatment, 10′–10 min, 20′–20 min, 30′–30 min.

We were interested if there is a possible protective effect of lutein in BV-2 microglial cells against H_2_O_2_-induced cytotoxicity. The cells were treated or 24 h pre-treated with 7.5 and 10 ng/µL of lutein, then 5 or 10 µM of H_2_O_2_ was added to the cells. We determined the ROS in BV-2 cells after addition of H_2_O_2_ in 10 to 30 min. Both 5 and 10 µM of H_2_O_2_ significantly elevated the ROS in the BV-2 cells compared to controls. Besides, 10 µM of H_2_O_2_ was more effective on ROS production (Figure 1). Amounts of 7.5 and 10 ng/µL of freshly added lutein significantly decreased the ROS in the cells after 5 and 10 µM of H_2_O_2_ treatments (Figure 1). Lutein itself did not affect ROS production compared to controls. Although there was no significant difference detected between the pre-treatment and fresh lutein treatment in the intracellular ROS compared to controls, we found that the freshly added lutein was more successful in reducing ROS after the H_2_O_2_ treatment (Figure 1). As 10 µM of H_2_O_2_ induced the ROS more effectively, we continued to use this concentration for the further experiments.

### 3.2. Lutein Modifies the mRNA Expressions of Antioxidant Enzymes Catalase, Superoxide Dismutase 2 and Heme Oxygenase-1

H_2_O_2_ is formed in mammals as a short-lived product in biochemical processes with its principle physiological role to activate the signalling pathways, stimulate antioxidants and induce ROS generation. The production and accumulation of excessive H_2_O_2_ is harmful and toxic to cells. To prevent cellular and tissue damage, it must be removed rapidly and efficiently by converting into other, less dangerous substances. We examined the antioxidant effects of lutein on H_2_O_2_-activated BV-2 microglia by profiling selected antioxidant enzymes, which were encoded by genes related to oxidative stress by real-time PCR. We determined the relative mRNA expression of catalase (*CAT*), superoxide dismutase 2 (*SOD2*) and heme oxygenase-1 (*HO-1*) in H_2_O_2_-induced oxidative stress conditions alone or in the presence of lutein.

**Figure 2:**
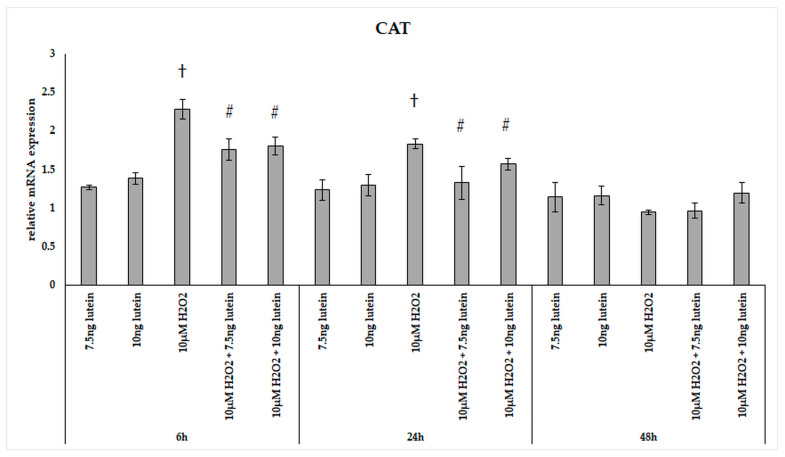
Relative mRNA expression of catalase in lutein and in H_2_O_2_-treated BV-2 cells. The columns represent the mean values and error bars indicate the standard deviation (SD) of three independent experiments (*n* = 3), each performed in triplicate, and are presented relative to own control cells. † represents statistical significance of H_2_O_2_-treated cells compared to controls, # marks statistical significance of combined (lutein with H_2_O_2_) treatments compared to H_2_O_2_ treatments; the level of significance was *p* < 0.05. Real-time PCR was performed with the SYBR green protocol using gene-specific primers. β-actin was used as house-keeping gene for the normalization. The relative expression of controls was regarded as 1.

In our experiments, we did not observe significant changes in the expression of catalase in the lutein treated cultures (Figure 2). The relative mRNA expression of catalase was significantly induced by 10 µM H_2_O_2_ both at 6 and 24 h cultures compared to controls, and the expression levels returned to the baseline levels at 48 h. The combined lutein and H_2_O_2_ treated cells showed a slighter increase suggesting that lutein decreased the effect of H_2_O_2_ (Figure 2).

**Figure 3:**
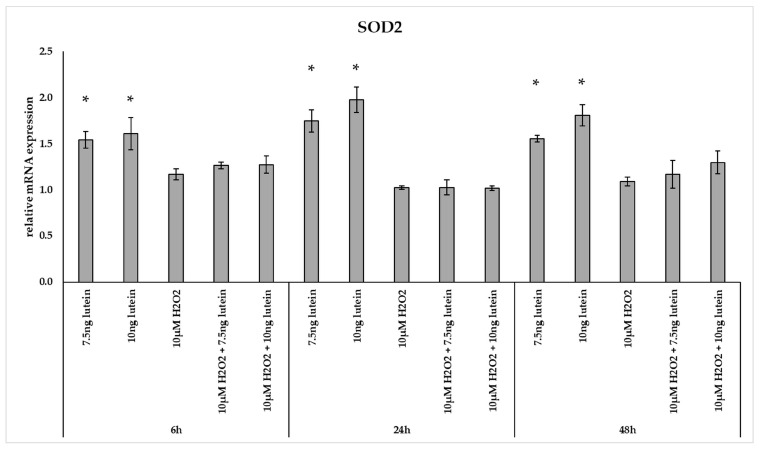
Relative mRNA expression of SOD2 in the lutein and in H_2_O_2_ treated BV-2 cells. The columns represent the mean values and error bars indicate the standard deviation (SD) of three independent experiments (*n* = 3), each performed in triplicate, and are presented relative to own control cells. * indicates statistical significance of lutein treatments versus controls; the level of significance was *p* < 0.05. Real-time PCR was performed with the SYBR green protocol using gene-specific primers. β-actin was used as house-keeping gene for the normalization. The relative expression of controls was regarded as 1.

Interestingly, the relative mRNA expression of *SOD2* significantly increased in the 7.5 and 10 ng/µL of lutein treated cells at 6, 24 and 48 h compared to appropriate controls (Figure 3). H_2_O_2_ alone and co-cultured with lutein did not alter significantly the relative mRNA expression of *SOD2*.

**Figure 4:**
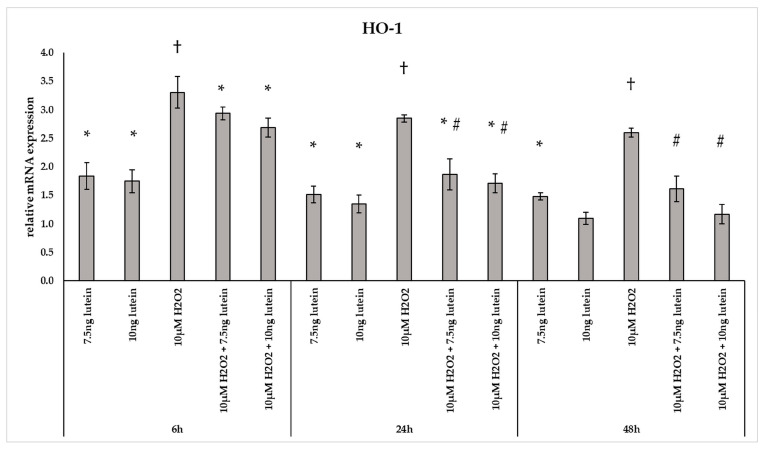
Relative mRNA expression of HO-1 in lutein and in H_2_O_2_-treated BV-2 cells. The columns represent the mean values and error bars indicate the standard deviation (SD) of three independent experiments (*n* = 3), each performed in triplicate, and are presented relative to own control cells. * represents statistical significance of lutein treatments versus controls; † shows statistical significance of H_2_O_2_-treated cells compared to controls; # indicates statistical significance of combined (lutein with H_2_O_2_) treatments compared to H_2_O_2_ treatments; the level of significance was *p* < 0.05. Real-time PCR was performed with the SYBR green protocol using gene-specific primers. β-actin was used as house-keeping gene for the normalization. The relative expression of controls was regarded as 1.

The phase II detoxifying enzyme HO-1 is a crucial component of the cellular stress response [29]. In our experiments H_2_O_2_ caused a 2–3-fold change in the mRNA expression of *HO-1* at all three examined time points (Figure 4). Culturing BV-2 cells with 7.5 and 10 ng/µL of lutein, we found a slight increase in the *HO-1* mRNA expression compared to appropriate controls at both 6 and 24 h. A gradual decrease was detected in the *HO-1* gene expression when lutein and H_2_O_2_ were added together to the cells compared to H_2_O_2_ (Figure 4).

It is known that oxidative stress upregulates expression of antioxidant enzymes [30]. The expression of *CAT* and *HO-1* was upregulated in H_2_O_2_ treated BV-2 cells. In the case of combined lutein with H_2_O_2_ treatments, the expressions remained lower, suggesting that lutein decreased the expression levels compared to H_2_O_2_ administration alone.

**Figure 5:**
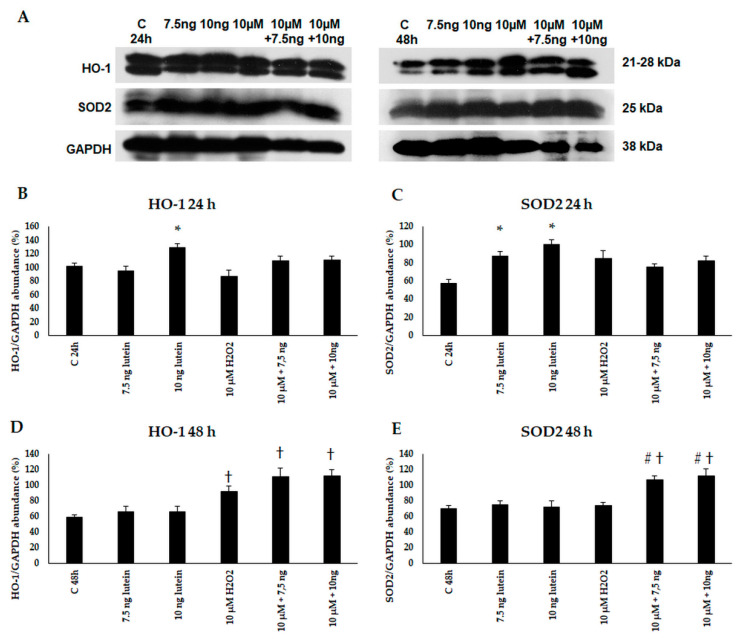
(**A**) Western blot analyses of antioxidant proteins HO-1 and SOD2 in lutein and in H_2_O_2_-treated BV-2 cells at 24 and 48 h. GAPDH was used as loading control. (**B**–**E**) Optical density analyses of HO-1 and SOD2 in BV-2 cells at 24 and 48 h. The columns represent mean values and error bars represent standard deviation (SD) of three independent determinations (*n* = 3). * indicates statistical significance of lutein treatments versus controls; † indicates statistical significance of H_2_O_2_ -treated cells compared to controls; # indicates statistical significance of combined (lutein with H_2_O_2_) treatments compared to H_2_O_2_ treatments; the level of significance was *p* < 0.05. Abbreviations: C marks the appropriate absolute controls at 24 and 48 h; 7.5 ng means 7.5 ng/µL of lutein; 10 ng means 10 ng/µL of lutein; 10 µM refers to 10 µM H_2_O_2_.

Next, we performed Western blot experiments to determine the HO-1 and SOD2 protein levels in BV-2 cells. An amount of 10 ng/µL of lutein increased the protein levels at 24 h. An amount of 10 µM of H_2_O_2_ treatment alone and in the presence of lutein induced HO-1 protein expression in BV-2 cells at 48 h. In case of SOD2, the protein levels increased at 24 h in the lutein-treated cells, and at 48 h in the complex-treated cells (Figure 5).

### 3.3. Lutein Increases Heme Content of BV-2 Cells

Iron is an indispensable element required by the cells for their normal functioning and plays role in maintaining the brain homeostasis. Microglia cells are also able to accumulate iron in inflammation, developmental processes, myelinization and neurodegenerative diseases.

**Figure 6:**
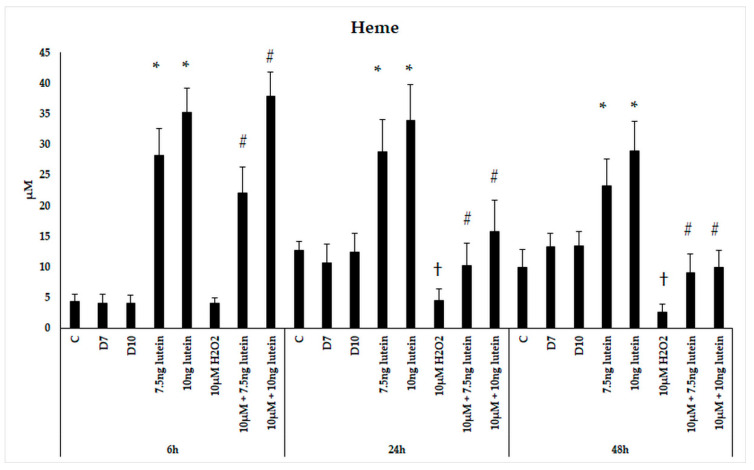
Determinations of heme concentration in lutein and H_2_O_2_-treated BV-2 cells. The columns represent the mean values and error bars indicate the standard deviation (SD) of four independent experiments (*n* = 4), each performed in triplicate, and are presented relative to own control cells. * indicates statistical significance of lutein treatments versus controls; † indicates statistical significance of H_2_O_2_-treated cells compared to controls; # indicates statistical significance of combined (lutein with H_2_O_2_) treatments compared to H_2_O_2_ treatments; the level of significance was *p* < 0.05. Abbreviations: C marks the appropriate absolute controls at 6, 24 and 48 h; D7 means the same amount of DMSO as 7.5 ng/µL of lutein was added; D10 means the same amount of DMSO as 10 ng/µL of lutein was used in the treatments.

Since *HO-1* is involved in antioxidant processes and the degradation of heme, we performed measurements on the intracellular heme concentration following administrations of lutein and H_2_O_2_. Microglial cells treated with lutein showed a significant elevation in heme content (Figure 6). The concentration of heme in BV-2 cells increased in accordance of the lutein concentrations and decreased when the cells were treated with H_2_O_2_. In the combined lutein and H_2_O_2_ treated cells, the heme concentration showed a time dependent decreasing tendency.

**Figure 7:**
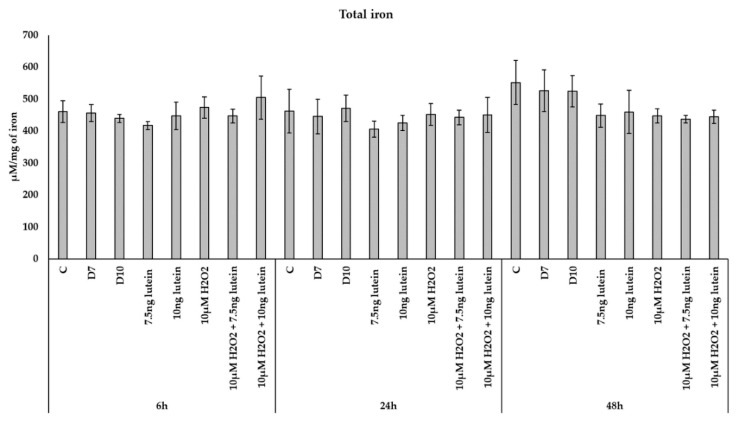
Determination of the total iron content in lutein and in H_2_O_2_-treated BV-2 cells. The columns represent the mean values, and error bars indicate the standard deviation (SD) of three independent experiments (*n* = 3), each performed in triplicate, and are presented relative to own control cells. Abbreviations: C marks the appropriate absolute controls at 6, 24 and 48 h; D7 means the same amount of DMSO as 7.5 ng/µL of lutein was added; D10 signs the same amount of DMSO as 10 ng/µL of lutein was used in the treatments.

We were also interested in if the iron content of BV-2 cells was altered, therefore we determined the total iron content of microglia as a result of the previous treatments. We did not detect changes in total iron content of the BV-2 cells in the lutein or H_2_O_2_ treatments nor in combinations of the former two (Figure 7).

### 3.4. Lutein Modulates the Expression of Genes Involved in Mitochondrial Iron Metabolism under Normal and H_2_O_2_-Induced Stress Conditions in BV-2 Cells

Since the total iron content showed no significant alteration after the different treatments, while the level of heme was elevated due to lutein administration, we investigated whether there was a rearrangement of intracellular iron.

We examined the expression of four genes that are involved in mitochondrial iron uptake and utilization: mitoferrin 2 (*MFRN2*), ferrochelatase (*FECH*), mitochondrial cysteine desulfurase (*NFS1*) and mitochondrial ferritin (*FTMT*). MFRN2 is a ubiquitously expressed iron transporter on the mitochondrial inner membrane and is responsible for mitochondrial import of Fe^2+^ from the intermembrane space to the matrix [15]. Inside the organelle, iron is incorporated into heme and Fe-S clusters or can be stored in FTMT.

**Figure 8:**
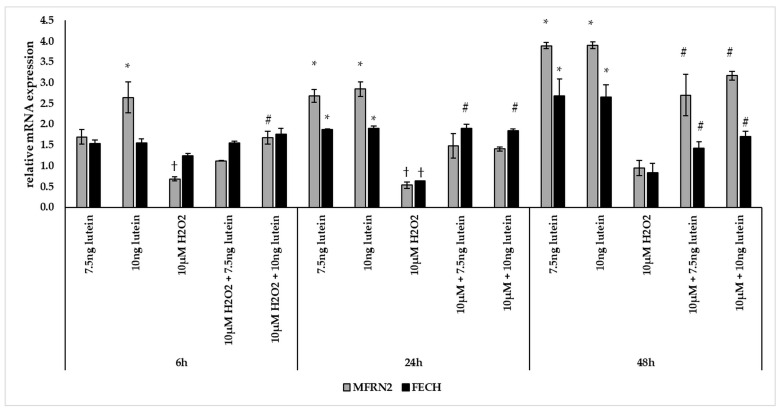
Effects of lutein and H_2_O_2_ treatment on mRNA expressions of MFRN2 and FECH. The columns represent the mean values, and error bars indicate the standard deviation (SD) of three independent experiments (*n* = 3), each performed in triplicate, and are presented relative to own control cells. * indicates statistical significance of lutein treatments versus controls; † indicates statistical significance of H_2_O_2_-treated cells compared to controls; # indicates statistical significance of combined (lutein with H_2_O_2_) treatments compared to H_2_O_2_ treatments; the level of significance was *p* < 0.05. Real-time PCR was performed with the SYBR green protocol using gene-specific primers. β-actin was used as house-keeping gene for the normalization. The relative expression of controls was regarded as 1.

BV-2 cells treated with lutein showed significantly increasing *MFRN2* mRNA expression compared to appropriate controls (Figure 8). The addition of 10 µM H_2_O_2_ decreased the *MFRN2* expression significantly both at 6 and at 24 h compared to controls. The expression of *MFRN2* increased in the combined lutein and 10 µM H_2_O_2_ treated cells after 48 h compared to H_2_O_2_ treated cells.

The mRNA expression of *FECH* was elevated via lutein treatment at 24 and 48 h compared to appropriate controls (Figure 8). Upon H_2_O_2_ treatment, the mRNA expression significantly decreased at 24 h compared to control and showed no alterations at 6 and 48 h. In the combined lutein with H_2_O_2_ treatments, we detected elevation at 24 and 48 h compared to H_2_O_2_ (Figure 8).

**Figure 9:**
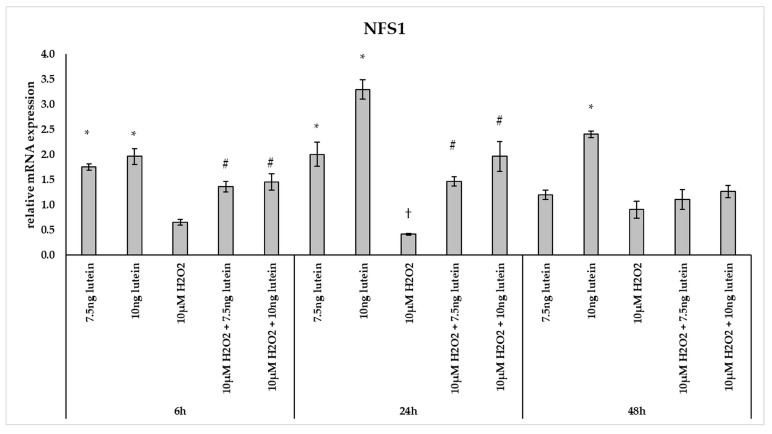
Effects of lutein and H_2_O_2_ on NFS1 mRNA expression. The columns represent the mean values, and error bars indicate the standard deviation (SD) of three independent experiments (*n* = 3), each performed in triplicate, and are presented relative to own control cells. * marks statistical significance of lutein treatments versus controls; † indicates statistical significance of H_2_O_2_-treated cells compared to controls; # represents statistical significance of combined (lutein with H_2_O_2_) treatments compared to H_2_O_2_ treatments; the level of significance was *p* < 0.05. Real-time PCR was performed with the SYBR green protocol using gene-specific primers. β-actin was used as house-keeping gene for the normalization. The relative expression of controls was regarded as 1.

Furthermore, we investigated the *NFS1* mRNA expression, which participates in Fe-S cluster assembly providing sulfur from cysteine. The 7.5 and 10 ng/µL of lutein treatments increased the *NFS1* expression at a significantly higher level at 6 and 24 h, and also in the case of 10 ng/µL of lutein at 48 h compared to the appropriate controls (Figure 9). Treatment with 10 µM H_2_O_2_ decreased the expression of *NFS1* at 24 h, while it showed no alterations at 6 and 48 h. In the combined treatment a significant increase was observed at 6 and 24 h compared to H_2_O_2_ treatments (Figure 9).

**Figure 10:**
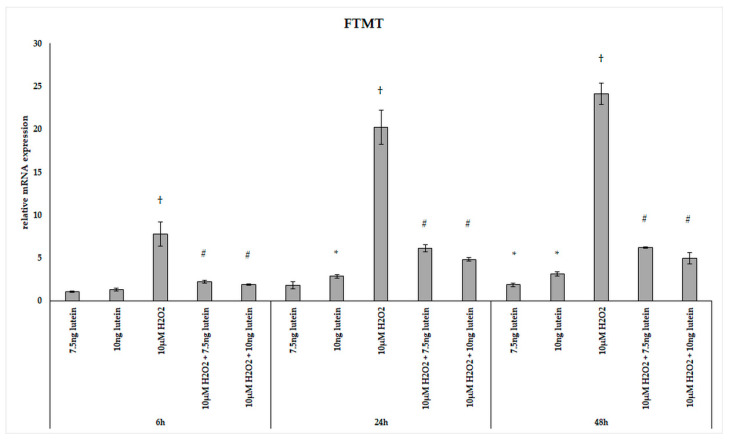
Effects of lutein and H_2_O_2_ on FTMT mRNA expression. The columns represent the mean values, and error bars indicate the standard deviation (SD) of three independent experiments (*n* = 3), each performed in triplicate, and are presented relative to own control cells. * indicates statistical significance of lutein treatments versus controls; † indicates statistical significance of H_2_O_2_-treated cells compared to controls; # indicates statistical significance of combined (lutein with H_2_O_2_) treatments compared to H_2_O_2_ treatments; the level of significance was *p* < 0.05. Real-time PCR was performed with the SYBR green protocol using gene-specific primers. β-actin was used as house-keeping gene for the normalization. The relative expression of controls was regarded as 1.

FTMT, a H-ferritin-like protein, with a specific mitochondrial location has been identified as a modulator of cellular iron metabolism through having a protective effect by storing free iron. We determined the relative expression of *FTMT* followed the above treatments of BV-2 cells. Adding 7.5 ng/µL of lutein significantly increased the *FTMT* mRNA expression only at 48 h, while 10 ng/µL of lutein promoted *FTMT* expression both at 24 and at 48 h compared to appropriate controls (Figure 10). H_2_O_2_ treatment strongly elevated the expression of *FTMT* at each examined time points compared to controls. The combined treatments two compound together showed an increase throughout the whole experiment compared to H_2_O_2_-treated cells and also to controls (Figure 10).

Lutein induced the gene expression of the mitochondrial iron transporter *MFRN2*, the heme synthesis enzyme *FECH*, the cysteine desulfurase *NFS1* and the mitochondrial iron storing *FTMT*. H_2_O_2_ significantly decreased the expression of *MFRN2* at 6 and 24 h, *FECH* at 24 h, NFS1 at 24 h, while there was highly elevated *FTMT* expression at 6, 24 and 48 h. Lutein with H_2_O_2_ treatment increased the *FECH* at 24 and 48 h, the *MFRN2* at 48 h and the *NFS1* at 6 and 24 h compared to the H_2_O_2_ treatments. Lutein counteracted the effect of H_2_O_2_ on *NFS1* and *FTMT*.

**Figure 11:**
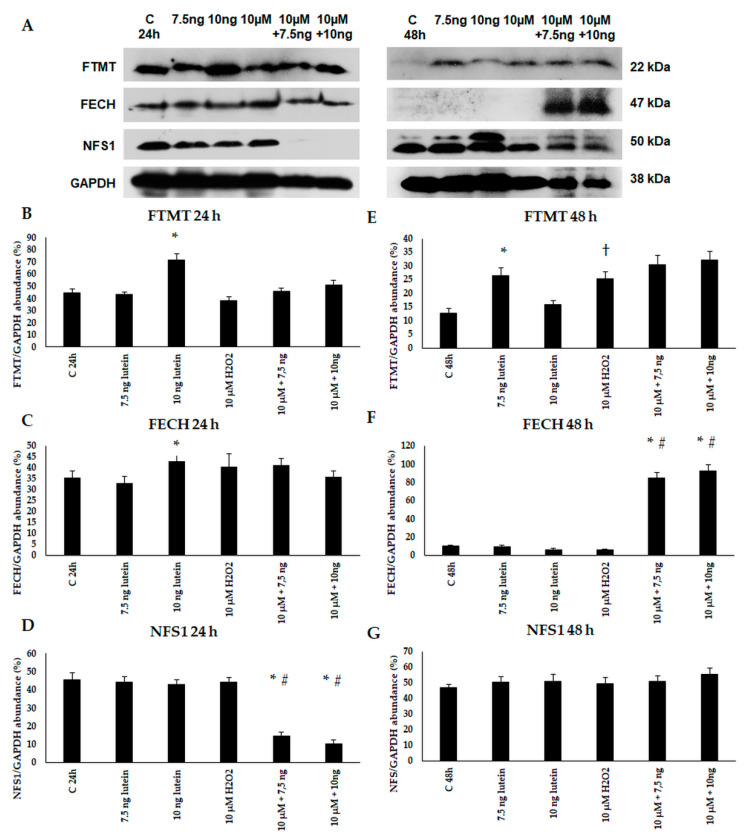
(**A**) Western blot analyses of mitochondrial proteins FTMT, FECH and NFS1 in lutein and in H_2_O_2_-treated BV-2 cells at 24 and 48 h. GAPDH was used as loading control. (**B**–**G**) Optical density analyses of FTMT, FECH and NFS1 in BV-2 cells at 24 and 48 h. The columns represent mean values and error bars represent standard deviation (SD) of three independent determinations (*n* = 3). * indicates statistical significance of lutein treatments versus controls; † indicates statistical significance of H_2_O_2_-treated cells compared to controls; # indicates statistical significance of combined (lutein with H_2_O_2_) treatments compared to H_2_O_2_ treatments; the level of significance was *p* < 0.05. Abbreviations: C marks the appropriate absolute controls at 24 and 48 h; 7.5 ng means 7.5 ng/µL of lutein; 10 ng means 10 ng/µL of lutein; 10 µM refers to 10 µM H_2_O_2_.

Thereafter, we performed Western blot experiments to determine the mitochondrial FTMT, FECH and NFS1 protein levels and changes in the lutein and H_2_O_2_-treated BV-2 cells. The expression of FTMT protein increased at 24 h in the 7.5 ng/µL of lutein-treated cells and at 48 h in the 10 ng/µL of lutein treatment. H_2_O_2_ increased the FTMT protein level at 48 h (Figure 11). FECH protein level was induced by 10 ng/µL of lutein at 24 h, and in the complex treatments at 48 h. We did not find significant alteration in NFS1 protein level, except in the complex treatments at 24 h.

### 3.5. Lutein Alters the Anti-Inflammatory and Pro-Inflammatory Cytokine Secretions in H_2_O_2_-Induced Stress in BV-2 Cells

**Figure 12:**
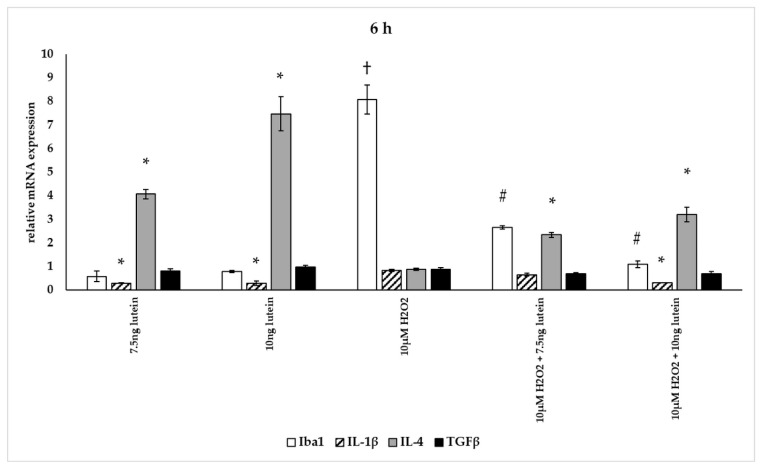
Effects of lutein and H_2_O_2_ on Iba1, IL-1β, IL-4 and TGFβ mRNA expression at 6 h. The columns represent the mean values, and error bars indicate the standard deviation (SD) of three independent experiments (*n* = 3), each performed in triplicate, and are presented relative to own control cells. * indicates statistical significance of lutein treatments versus controls; † indicates statistical significance of H_2_O_2_-treated cells compared to controls; # indicates statistical significance of combined (lutein with H_2_O_2_) treatments compared to H_2_O_2_ treatments; the level of significance was *p* < 0.05. Real-time PCR was performed with the SYBR green protocol using gene-specific primers. β-actin was used as house-keeping gene for the normalization. The relative expression of controls was regarded as 1.

**Figure 13:**
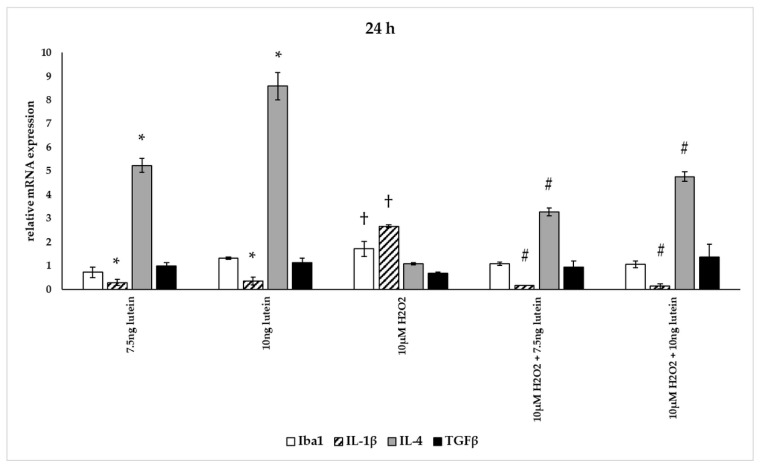
Effects of lutein and H_2_O_2_ on Iba1, IL-1β, IL-4 and TGFβ mRNA expression at 24 h. The columns represent the mean values, and error bars indicate the standard deviation (SD) of three independent experiments (*n* = 3), each performed in triplicate, and are presented relative to own control cells. * indicates statistical significance of lutein treatments versus controls; † indicates statistical significance of H_2_O_2_ treated cells compared to controls; # indicates statistical significance of combined (lutein with H_2_O_2_) treatments compared to H_2_O_2_ treatments; the level of significance was *p* < 0.05. Real-time PCR was performed with the SYBR green protocol using gene-specific primers. β-actin was used as house-keeping gene for the normalization. The relative expression of controls was regarded as 1.

**Figure 14:**
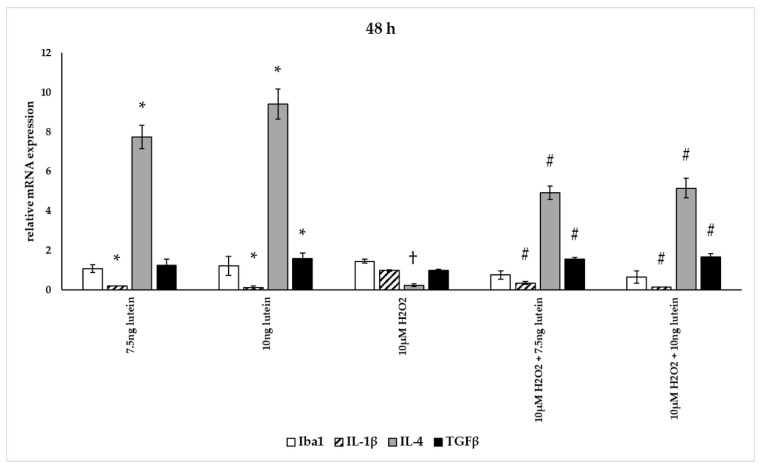
Effects of lutein and H_2_O_2_ on Iba1, IL-1β, IL-4 and TGFβ mRNA expression at 48 h. The columns represent the mean values, and error bars indicate the standard deviation (SD) of three independent experiments (*n* = 3), each performed in triplicate, and are presented relative to own control cells. * indicates statistical significance of lutein treatments versus controls; † indicates statistical significance of H_2_O_2_-treated cells compared to controls; # indicates statistical significance of combined (lutein with H_2_O_2_) treatments compared to H_2_O_2_ treatments; the level of significance was *p* < 0.05. Real-time PCR was performed with the SYBR green protocol using gene-specific primers. β-actin was used as house-keeping gene for the normalization. The relative expression of controls was regarded as 1.

In our measurements, lutein suppressed the induced ROS in BV-2 cells and altered antioxidant gene expression. ROS are known for mediating the redox balance, tissue homeostasis and inflammatory regulation. The imbalance between the production of ROS and their elimination by protective mechanisms can lead to chronic inflammation [31,32,33]. Therefore we were interested in the inflammatory changes, thus we further determined the effects of lutein on the cytokine secretion and expression under oxidative stress conditions in BV-2 cells. Iba1 and IL-1β expression were used as M1 markers, while IL-4 and TGFβ mRNA expression were determined as M2 phenotype markers (Figure 12, Figure 13 and Figure 14) [34]. The anti-inflammatory and antioxidant cytokine IL-10 and the pro-inflammatory and pro-oxidant marker TNFα were detected in the culture media of the differently treated cells. Both cytokines play important role in the regulation of inflammation, as well as in the immune responses and in controlling microglial activation in CNS [35,36].

The addition of lutein resulted in strong downregulation of IL-1β associated with pro-inflammatory response to H_2_O_2_. H_2_O_2_-induced the IL-1β mRNA expression in a time-dependent manner: it decreased at 6 h, increased at 24 h and returned to baseline at 48 h experiments. Lutein alone also decreased the expression of IL-1β in 6, 24 and 48 h experiments and in H_2_O_2_-treated BV-2 cells (Figure 12, Figure 13 and Figure 14).

Lutein alone and in the presence of H_2_O_2_ increased the anti-inflammatory cytokine IL-4 expression (Figure 12, Figure 13 and Figure 14).

Lutein seems to reduce M1 Iba1 marker, and in the 48 h treatments M2-specific TGFβ mRNA expression begins to increase, showing time dependence and suggesting that lutein attenuates the pro-inflammatory activation state of BV-2 cells.

**Figure 15:**
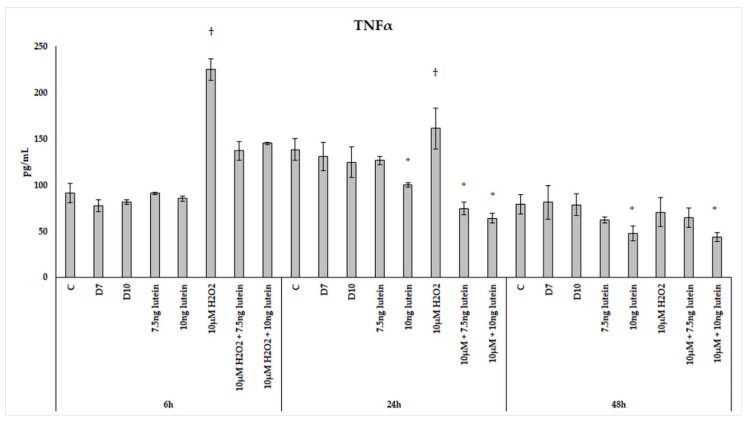
TNFα secretion in lutein and in H_2_O_2_-treated BV-2 cells. The columns represent the mean values, and error bars indicate the standard deviation (SD) of three independent experiments (*n* = 3), each performed in triplicate, and are presented relative to own control cells. * indicates statistical significance of lutein treatments versus controls; † indicates statistical significance of H_2_O_2_-treated cells compared to controls; the level of significance was *p* < 0.05. Abbreviations: C marks the appropriate absolute controls at 6, 24 and 48 h; D7 means the same amount of DMSO as 7.5 ng/µL of lutein was added; D10 means the same amount of DMSO as 10 ng/µL of lutein was used in the treatments.

We found that H_2_O_2_ treatment significantly elevated TNFα secretion of the microglia at 6 and 24 h (Figure 15). In the combined lutein with H_2_O_2_-treated cells, a decrease was observed in the pro-inflammatory TNFα secretion. A higher concentration of lutein alone showed a slight decrease in the measured TNFα concentrations at 24 and 48 h, but after the combined (lutein with H_2_O_2_) treatments, BV-2 cells significantly decreased TNFα secretion compared to the H_2_O_2_ treatment. The TNFα levels dropped close to the control levels at 6 h and further decreased at 24 h. In the 48 h cultures, TNFα concentration returned to the baseline in the cells; the effect of hydrogen peroxide appeared to have subsided.

**Figure 16:**
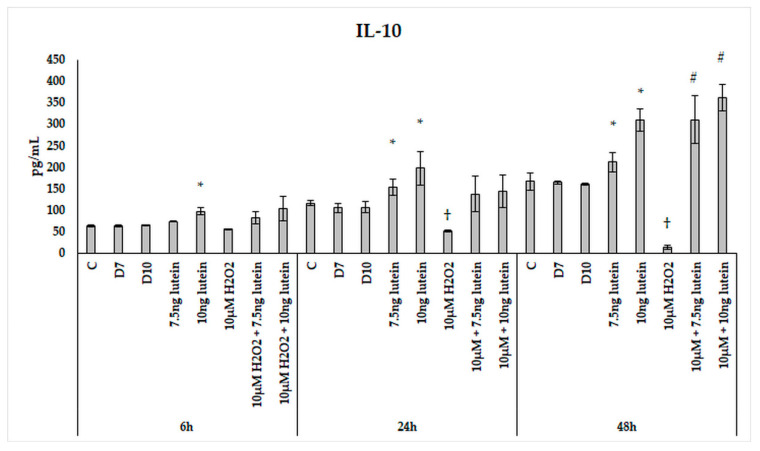
IL-10 secretion in lutein and in H_2_O_2_-treated BV-2 cells. The columns represent the mean values, and error bars indicate the standard deviation (SD) of three independent experiments (*n* = 3), each performed in triplicate, and are presented relative to own control cells. * indicates statistical significance of lutein treatments versus controls; † indicates statistical significance of H_2_O_2_-treated cells compared to controls; # indicates statistical significance of combined (lutein with H_2_O_2_) treatments compared to H_2_O_2_ treatments; the level of significance was *p* < 0.05. Abbreviations: C marks the appropriate absolute controls at 6, 24 and 48 h; D7 means the same amount of DMSO as 7.5 ng/µL of lutein was added; D10 signs the same amount of DMSO as 10 ng/µL of lutein was used in the treatments.

Upon H_2_O_2_ addition, the IL-10 secretion showed attenuation over time. There was a slight decrease after 24 h, and a significant decrease was observed in the 48 h cultures compared to the controls (Figure 16). Lutein increased IL-10 cytokine secretion in a time-dependent manner with an expressive elevation at 48 h. H_2_O_2_ decreased the secretion of IL-10 in the 24 and 48 h experiments. When the cells were co-cultured with lutein and H_2_O_2_, the secreted IL-10 was further increased compared to the lutein-treated cells, suggesting that oxidative stress triggers the protective effect of lutein.

We revealed that after lutein treatment, an increased anti-inflammatory IL-10 cytokine and a decreased pro-inflammatory TNFα cytokine secretion were detected in BV-2 microglia cells, suggesting that lutein also exerts an anti-inflammatory effect.

## 4. Discussion

Lutein is a xanthophyll carotenoid synthetized by plants. It has been well studied and widely used in supplements with a major role in protection against age-related eye tissue deterioration and degenerative diseases such as macular degeneration and cataracts [2]. The effectiveness of lutein supplementation is attributed largely to lutein’s antioxidant properties, which mean mainly the absorption of light-induced ROS [37]. Lutein has also been confirmed to protect skin from light-generated damage, to prevent cardiovascular diseases caused by hardening of the arteries and coronary heart disease and other diseases caused by aging. Meanwhile, lutein is often referred to as an antioxidant agent and lately linked to improvement in cognitive functions; however, the underlying mechanisms in other CNS regions are yet to be defined [38]. Microglia play a fundamental role in host defence, repair and homeostasis in the CNS [39]. Although H_2_O_2_ is not free radical, it diffuses freely and acts as a weak oxidizing and reducing agent. It is reported to generate free radicals as the ancestor of many other ROS [18]. The effect of lutein on H_2_O_2_-induced stress conditions in BV-2 microglia cell has not been investigated yet. In our study we focused on the effect of lutein on exogenous H_2_O_2_-induced stress related to ROS production, together with the possible antioxidant and anti-inflammatory responses in murine microglial cell line BV-2.

Oxidative stress take part in several metabolic and neurodegenerative disorders [40,41]. The production and the accumulation of excessive H_2_O_2_ is harmful and toxic to cells through the oxidation of proteins, membrane lipids and DNA by the peroxide ions [42,43]. We determined the effect of lutein on the ROS generation in H_2_O_2_-induced oxidative stress. H_2_O_2_ significantly elevated the ROS in the BV-2 cells. ROS was directly proportional to the concentration of H_2_O_2_. Lutein alone did not affect ROS production compared to controls, but the addition of lutein significantly decreased the ROS in the H_2_O_2_-treated cells. Experimental evidence suggests that ROS generation plays a critical role in the regulation of the fate of microglia, such as proliferation, apoptosis induction, neurotransmitter releasing and production of pro-inflammatory cytokines. High ROS concentrations overrule the microglia cellular repair mechanism for minor oxidative stress and lead to oxidative damage to proteins and nucleic acids with subsequent risk for neuronal populations [9,44]. Based on our results, lutein provided protection for BV-2 cells against H_2_O_2_-induced oxidative stress.

Despite the fact that the brain has a high rate of oxidative metabolism, the CNS has low levels of antioxidants and is vulnerable to oxidative stress. The activities of the enzymes catalase, SOD and glutathione peroxidase have pivotal roles in the maintenance of redox balance in both microglia and astrocytes [9,45,46]. Catalase is one of the essential antioxidant enzymes which alleviate oxidative stress by destroying cellular H_2_O_2_, converting it to oxygen and water. Its activity is stimulated by H_2_O_2_ [18]. In the H_2_O_2_-induced oxidative stress mechanism, the concentration of H_2_O_2_ falls due to the action of catalase [47]. In accordance with literary knowledge, in our experiments the gene expression of *CAT* was upregulated after H_2_O_2_ treatment and showed a decreasing tendency with time. The mRNA level of *CAT* returned to the baseline at 48 h, and the effect of H_2_O_2_ was debilitated in *CAT* gene expression. However, in case of lutein treatment, we did not detect any changes in the expression of *CAT*; in the case of cells treated with the combination of lutein and H_2_O_2_, a decrease was observed in the mRNA level of the enzyme compared to H_2_O_2_ treatments. This observation suggests that lutein reduced the effect of H_2_O_2_.

SOD2 is a key component of antioxidant defence system against mitochondrial superoxide radicals [48]. SOD2 together with the two other isoforms (SOD1, SOD3) is able to protect cells and tissues from ROS generated by both endogenous and exogenous sources [48,49]. SOD2 is among the endogenous antioxidant enzymes which converts ROS to H_2_O_2_, which is ultimately converted into water by CAT and glutathione peroxidase [50]. H_2_O_2_ has been demonstrated to regulate the expression of SOD2 protein levels in a concentration-dependent manner, while the mRNA expression levels showed no difference in PC12 pheochromocytoma cells [51]. Primary microglia, induced by LPS showed increased *SOD2* gene expression, while the protein expression was not affected [52]. Downregulation of SOD2 was reported in chronic oxidative stress in aged endothelial vessels [53]. Fresta et al. has demonstrated that the inflammatory stimulus had not altered the expression of *SOD2* [54]. In our experiments, H_2_O_2_ had not altered the relative mRNA expression of *SOD2*. This result may be due to the fact that SOD2 convers superoxide radicals into H_2_O_2_ and not the other way around. Another explanation could be the increased expression of *CAT* that was shown to inhibit the expression of *SOD2* [48].

Upon lutein treatment, we detected an upregulation of both *SOD2* mRNA and protein expressions in BV-2 cells. Expression of *SOD2* is highly regulated, the low *SOD2* efficacy can increase superoxide anion levels, while high *SOD2* efficiency is able to increase H_2_O_2_ levels [48]. Lutein was reported to modify antioxidant enzymes in ARPE19 retinal pigment epithelium cells via increasing the *SOD1* and *SOD2* mRNA expressions [55]. SOD2 is known to have an important role in defence mechanisms that help cell survival under normal and stress conditions [52,56]. Increasing SOD2 was expected to support or improve neurons, while a dysregulation or mutation of the gene was reported in neurodegenerative disorders [57]. SOD2 was reported to prevent apoptosis in mice [58]. We suggest that the upregulation of antioxidant *SOD2* gene expression upon lutein treatment may contribute to protection for BV-2 cells.

HO-1 is a major mammalian stress-inducible ubiquitously expressed enzyme which is capable of modifying both innate and adaptive immune responses through modulating activation, differentiation and maturation, in addition to altering the polarization of several immune cells [59]. HO-1 protein expression was reported to be increased by LPS-induced inflammation in BV-2 microglial cells. It is known to have a protective function against inflammation [60]. Its transcription can be induced by iron and oxidative stress. Our results showed that H_2_O_2_ treatment caused a 2–3-fold increase in the mRNA expression of *HO-1* at all three time points examined. On the other hand, lutein slightly increased *HO-1* expression with a decreasing tendency in time. The combined treatment of lutein and H_2_O_2_ resulted in a lower expression compared to H_2_O_2_ treatment alone, suggesting that lutein relieves the stress induced by H_2_O_2_. The phase II detoxifying enzyme HO-1 is a crucial component of the cellular stress response [29]. It is known that oxidative stress upregulates expression of antioxidant enzymes [30]. The expression of *CAT* and *HO-1* was upregulated upon H_2_O_2_ in BV-2 cells. In the case of a treatment containing both lutein and H_2_O_2_, the expressions remained lower compared to H_2_O_2_, suggesting that lutein might be able to decrease or modulate the H_2_O_2_-induced oxidative stress. At protein level, HO-1 showed elevation upon lutein treatment and in the complex treatments, suggesting that there was a delay between mRNA and protein expressions.

The rate of oxidative stress and the regulation of HO-1 might be an important factor in the development of new treatment strategies for neurological diseases [59,61]. HO-1 expression is rapidly induced following acute brain injury in rat glia cells [62]. In response to oxidative stress, high expression of *HO-1* enhances the destruction of heme. The HO system oxidatively cleaves heme, and as a result the released ferrous iron can trigger ferritin synthesis for iron storage as well as promote ferroportin expression for iron export. Both phenomena help to avoid the increase in the labile iron pool and iron mediated toxicity via recycling iron to maintain the homeostasis in the cell. Elimination of the reactive heme iron by HO-1 involves cleavage of the protoporphyrin IX ring of heme with production of biliverdin and CO as well as the release of iron. All three end products of heme catabolism are cytoprotective and antioxidant [63,64].

Our results unveiled an interesting phenomenon that lutein strongly elevated the heme content of BV-2 cells. Heme concentration of BV-2 cells increased due to the lutein treatments, while decreased by H_2_O_2_ treatment. In the combined lutein and H_2_O_2_-treated cells, the heme concentration showed a time-dependent decreasing tendency compared to lutein-treated cells. Heme is a component of certain iron-containing proteins, including hemoglobin, myoglobin and neuroglobin [65]. Heme is essential for many biological functions, and as a prosthetic group it also plays important catalytic roles among others in peroxidases and H_2_O_2_ detoxification via catalase and in nitric oxide generation [15,66]. The bioavailability of heme is tightly controlled, regardless of the source for synthesis; the transport and traffic of it must be a highly regulated and safe process in each subcellular compartment [66].

Recycling iron from heme by HO-1 is crucial in maintaining iron homeostasis [26]. In this context, we determined if there are changes in total iron content of microglia, and we did not detect significant alterations in total iron content of BV-2 cells in lutein or H_2_O_2_ treatments, nor in combinations of the former substances. Since the total iron content did not change in our measurements, while the level of heme was elevated due to lutein treatment, iron appeared to remain in BV-2 cells and either integrated into other proteins or was stored. The control of iron homeostasis within microglia is not completely defined; however, microglia and iron deposits accumulate at the site of damage in many neurodegenerative diseases. Whether these accumulations are the cause or the consequence of the disease is currently unknown [67].

Mitochondria have a major role not only in the regulation of redox processes and maintaining the energy state of the cells, but also as pivotal centres of intracellular iron metabolism. The majority of the enzymes for Fe-S cluster synthesis and Ferrochelatase, the enzyme incorporating iron into heme, are located in the mitochondria. The Mitoferrin transporters (Mitoferrin 1 and 2) are located on the mitochondrial inner membrane and are liable for the mitochondrial import of Fe^2+^ from the intermembrane space to the matrix [68]. MFRN2 mediates iron uptake and is likely required for heme synthesis of hemoproteins and Fe-S cluster assembly in non-erythroid cells. The iron delivered into the mitochondria, probably as ferrous iron, is then presumably transferred to FECH to catalyse ferrous iron incorporation into Protoprophyrin IX to produce heme [69]. The substrates of Ferrochelatase, both ferrous iron and Protoporphyrin IX, are potentially toxic to the cell, thus they required delivery directly to FECH [70,71].

Our results revealed that lutein induced the expression of mitochondrial iron importer *MFRN2* in BV-2 cells, while the level of *MFRN2* decreased with H_2_O_2_ treatments and slightly increased in the combination-treated cultures. These results suggest that lutein has an effect on the iron transport from the cytoplasm towards into mitochondria. In our experiments H_2_O_2_ decreased the gene expression of *FECH* and increased ROS, while lutein induced an elevated expression of *FECH* and decreased ROS. At protein level, lutein and H_2_O_2_ showed a slight increase in FECH while later only the complex treatment was able to elevate the FECH protein level.

In our experiments, H_2_O_2_ increased the *HO-1* expression and decreased the heme concentration, while lutein increased the heme, therefore we examined whether these changes could affect mitochondrial iron storage. The released iron from heme may be stored in the mitochondria and also might be incorporated into Fe-S clusters. The mitochondrion also possesses iron storage capacity in non-toxic form by FTMT, which supplies protection against oxidative damage, while a high amount of accumulated and stored iron can induce iron overload and toxicity. This mitochondrial overload was observed in diseases with specific genetic impairments and neurodegenerative disorders, among others, such as Alzheimer’s Disease, Parkinson’s Disease and sideroblastic anemia [26,72,73,74]. We found that H_2_O_2_ treatment induced mitochondrial FTMT expression. Overexpression of FTMT leads to the partitioning of iron away from Fe-S cluster and heme synthesis [72]. The increased expression of *FTMT* in H_2_O_2_-treated BV-2 cells suggests the defend mechanisms of mitochondrion. Lutein treatment increased the expression of mitochondrial iron storage FTMT protein suggesting that lutein triggers iron accumulation in the mitochondria. H_2_O_2_ induced FTMT protein levels at 48 h.

The product of Fe-S or heme biosynthesis is involved in overall regulation of mitochondrial iron homeostasis [74]. In our experiments, lutein increased the expression of the cysteine desulfurase coding *NFS1*, while H_2_O_2_ decreased it, and in combined lutein and H_2_O_2_ treatment we observed a less pronounced increase compared to lutein alone, suggesting that lutein suppressed the effect of H_2_O_2_. We did not observe significant alteration in NFS1 protein levels, except in the combined treatments at 24 h. NFS1 is required for the Fe-S cluster assembly by providing sulfur [25]. There is a wide range of biological and biochemical functions provided by Fe-S clusters including electron transfer, redox catalysis, non-redox catalysis, protein synthesis, ATP production, oxidative stress defence and preservation of genome integrity [25]. H_2_O_2_ was reported to promote disassembly of Fe-S clusters in HeLa cells and suggested that assembly and disassembly processes of Fe-S clusters were highly dynamic and sensitive for oxidative stress [75]. Although, lutein seems to elevate iron storage in the mitochondria, it does not affect iron-sulfur cluster synthesis, but it triggers FECH synthesis suggesting its role in the promotion of heme synthesis.

Mitochondria, in addition to storage, incorporate iron into heme and Fe-S clusters through their synthesis. The products are either transferred within mitochondria or are exported into the cytoplasm [76,77,78]. Based on our results, lutein seems affect the iron transport in BV-2 microglia by transporting directing it from the cytosol towards into mitochondria and within mitochondria induce heme synthesis and probably Fe-S cluster synthesis also.

Depending on the environmental stimuli, microglia mediate both protective and harmful responses to brain injuries, and thus are the major immune response regulators in neurodegenerative diseases [32]. ROS are suggested as mediators of microglial activation, although the underlying mechanisms of microglial activation are not completely understood yet [79]. In our study we triggered oxidative stress in BV-2 microglia with H_2_O_2_. The microglial phenotypes are tightly regulated by levels of ROS. In responses to inflammatory effects the microglia are activated and polarize toward an M1-phenotype, thus the cells release highly pro-inflammatory cytokines such as TNFα, IL-1, IL-6 or IL-12. An increased pro-inflammatory microglia phenotype cytokine expression has been observed in neurodegenerative diseases. The M2-phenotype microglia produce and release high levels of anti-inflammatory cytokines including IL-4, IL-10 and IL-13; in parallel, they express low inflammatory cytokines [9,32]. It has been revealed that lutein also possesses anti-inflammatory properties [3]. Moreover, lutein has been implicated in attenuating the inflammatory responses by the regulation of pro-inflammatory mediators and cytokine syntheses in lipopolysaccharide-induced inflammation in BV-2 microglia [80]. In our experiments the BV-2 cells secreted a low amount of both anti-inflammatory IL-10 and pro-inflammatory TNFα cytokines in the controls. In response to H_2_O_2_-induced stress stimuli, the cells released increased amount of TNFα and decreased IL-10. We demonstrated that lutein alone as well as together with H_2_O_2_ treatment was able to upregulate IL-10 levels, without the upregulation of pro-inflammatory molecules in BV-2 cells. In the lutein and H_2_O_2_-treated cells, the TNFα cytokine levels remained at a lower level compared to cells which received H_2_O_2_ alone, with a time dependent declining trend [81]. Recently, IL-10 has been reported to change macrophage function by assisting the clearance of damaged mitochondria and modifying cellular metabolism to inhibit inflammation [82]. The elevation of IL-10 according to lutein treatment is proposed as the principal anti-inflammatory, inhibitory or self-regulating operation of the cytokine through autocrine and paracrine mechanisms [83]. Our results suggest that lutein might be able to shift the microglia polarization to the anti-inflammatory phase, although exploring the possibility with the undergoing mechanisms requires further experiments [9,84].

It was highlighted by several studies that an inverse correlation exists regarding the health benefits of antioxidants and free radicals [85]. The antioxidant can delay the process of aging by scavenging free radical species. Lutein has been reported to have higher antioxidant efficacy than other common carotenoids [86]. Taken together, our results reveal that lutein suppressed ROS in BV-2 microglia under H_2_O_2_-induced stress conditions. Lutein increased the cell viability compared to H_2_O_2_ treatments. Lutein induced the expression of antioxidant *SOD2* and decreased the H_2_O_2_-induced *HO-1* expression in BV-2. Microglial cells treated with lutein showed a significant elevation in heme content without total iron alterations. Lutein elevated the anti-inflammatory IL-10 cytokine secretion of the BV-2 cells, and in presence of H_2_O_2_ it decreased the pro-inflammatory TNFα levels.

These results suggest that upon lutein treatment, BV-2 cells are highly protected against oxidative stress on several fronts: reducing ROS, elevating the expression of antioxidant enzymes, changing the pattern of pro-and anti-inflammatory cytokine production, as well as altering the iron metabolism of the cells. The latter effect means that the lutein-treated cells increase the uptake of iron into the mitochondria, but instead of storing it in the mitochondria, iron is incorporated into products such as heme and iron-sulfur clusters. As mitochondrial-stored iron may worsen the harm of oxidative stress, this is a useful protection.

The imbalance between antioxidants and overproduced pro-oxidants together with the altered inflammatory conditions can lead towards the development of neurodegenerative disorders [32,35,36]. Unravelling the possible protective effects of lutein under stress conditions in microglia may help in preventing ROS in microglia activation-mediated neuronal degeneration in oxidative stress scenarios.

## Figures and Tables

**Table 1:** Real-time PCR gene primer sequence list.

Target Gene	Gene Accession Number	Sequence 5′ → 3′
*β-actin* forward	NM_007393.5	CTGTCGAGTCGCGTCCA
*β-actin* reverse		TCATCCATGGCGAACTGGTG
*CAT* forward	NM_009804.2	AGAGGAAACGCCTGTGTGAG
*CAT* reverse		GCGTGTAGGTGTGAATTGCG
*FECH* forward	NM_007998.8	ACCACAGGCAGCAGCTTAAA
*FECH* reverse		CCTGTCGATTGTGCTCCACT
*FTMT* forward	NM_026286.3	TTAGGTCCCCTACTGGCCTC
*FTMT* reverse		CCAGGTTGATTTGGCGGTTG
*HO-1* forward	NM_010442.2	GTCAAGCACAGGGTGACAGA
*HO-1* reverse		ATCACCTGCAGCTCCTCAAA
*Iba1* forward	NM_019467.3	GGAAAGTCAGCCAGTCCTCC
*Iba1* reverse		CATCACTTCCACATCAGCTTTTGA
*IL-1β* forward	NM_008361.4	TGCCACCTTTTGACAGTGATG
*IL-1β* reverse		TGATGTGCTGCTGCGAGATT
*IL-4* forward	NM_021283.2	CTCGAATGTACCAGGAGCCA
*IL-4* reverse		AGGACGTTTGGCACATCCAT
*TGFβ* forward	NM_011577.2	CTGCTGACCCCCACTGATAC
*TGFβ* reverse		AGCCCTGTATTCCGTCTCCT
*MFRN2* forward	NM_145156.1	CCACTGTCACCACGCACAT
*MFRN2* reverse		GCCTCCAACACGTTCCGATA
*NFS1* forward	NM_010911.2	GATTGGAGCTGATCCTCGGG
*NFS1* reverse		AGAACCTGGCCACTCCCTTA
*SOD2* forward	NM_013671.3	GAACAATCTCAACGCCACCG
*SOD2* reverse		GCTGAAGAGCGACCTGAGTT

## Data Availability

Data is contained within the article or supplementary material.

## Supplementary Materials

The following are available online at https://www.mdpi.com/2076-3921/10/3/363/s1, Figure S1: Cell viability determinations of the lutein-treated BV-2 cells, Figure S2: Cell viability determinations of the H_2_O_2_-treated BV-2 cells, Table S1: Cell viability determinations of the lutein with H_2_O_2_-treated BV-2 cells.

## References

[1] Maoka T. (2020). Carotenoids as natural functional pigments. J. Nat. Med..

[2] Koushan K., Rusovici R., Li W., Ferguson L.R., Chalam K.V. (2013). The role of lutein in eye-related disease. Nutrients.

[3] Buscemi S., Corleo D., Di Pace F., Petroni M.L., Satriano A., Marchesini G. (2018). The effect of lutein on eye and extra-eye health. Nutrients.

[4] He R.R., Tsoi B., Lan F., Yao N., Yao X.S., Kurihara H. (2011). Antioxidant properties of lutein contribute to the protection against lipopolysaccharide-induced uveitis in mice. Chin. Med..

[5] Zhang Z.W., Xu X.C., Liu T., Yuan S. (2016). Mitochondrion-permeable antioxidants to treat ROS-burst-mediated acute diseases. Oxid. Med. Cell. Longev..

[6] Reuter S., Gupta S.C., Chaturvedi M.M., Aggarwal B.B. (2010). Oxidative stress, inflammation, and cancer: How are they linked?. Free Radic. Biol. Med..

[7] Bohn T. (2019). Carotenoids and markers of oxidative stress in human observational studies and intervention trials: Implications for chronic diseases. Antioxidants.

[8] Fiedor J., Burda K. (2014). Potential role of carotenoids as antioxidants in human health and disease. Nutrients.

[9] Rojo A.I., McBean G., Cindric M., Egea J., López M.G., Rada P., Zarkovic N., Cuadrado A. (2014). Redox control of microglial function: Molecular mechanisms and functional significance. Antioxid. Redox Signal..

[10] Yin J., Valin K.L., Dixon M.L., Leavenworth J.W. (2017). The role of microglia and macrophages in CNS homeostasis, autoimmunity, and cancer. J. Immunol. Res..

[11] Laffer B., Bauer D., Wasmuth S., Busch M., Jalilvand T.V., Thanos S., Meyer zu Hörste G., Loser K., Langmann T., Heiligenhaus A. (2019). Loss of IL-10 promotes differentiation of microglia to a M1 phenotype. Front. Cell. Neurosci..

[12] Burmeister A.R., Marriott I. (2018). The interleukin-10 family of cytokines and their role in the CNS. Front. Cell. Neurosci..

[13] Amor S., Puentes F., Baker D., Van Der Valk P. (2010). Inflammation in neurodegenerative diseases. Immunology.

[14] Harms A.S., Lee J.K., Nguyen T.A., Chang J., Ruhn K.M., Treviño I., Tansey M.G. (2012). Regulation of microglia effector functions by tumor necrosis factor signaling. Glia.

[15] Horowitz M.P., Greenamyre J.T. (2010). Mitochondrial iron metabolism and its role in neurodegeneration. J. Alzheimer’s Dis..

[16] Shimizu T., Nojiri H., Kawakami S., Uchiyama S., Shirasawa T. (2010). Model mice for tissue-specific deletion of the manganese superoxide dismutase gene. Geriatr. Gerontol. Int..

[17] Borrelli A., Schiattarella A., Bonelli P., Tuccillo F.M., Buonaguro F.M., Mancini A. (2014). The functional role of MnSOD as a biomarker of human diseases and therapeutic potential of a new isoform of a human recombinant MnSOD. Biomed. Res. Int..

[18] Nandi A., Yan L.J., Jana C.K., Das N. (2019). Role of catalase in oxidative stress- and age-associated degenerative diseases. Oxid. Med. Cell. Longev..

[19] Duvigneau J.C., Esterbauer H., Kozlov A.V. (2019). Role of heme oxygenase as a modulator of heme-mediated pathways. Antioxidants.

[20] Takahashi T., Shimizu H., Morimatsu H., Maeshima K., Inoue K., Akagi R., Matsumi M., Katayama H., Morita K. (2009). Heme oxygenase-1 is an essential cytoprotective component in oxidative tissue injury induced by hemorrhagic shock. J. Clin. Biochem. Nutr..

[21] Nnah I.C., Wessling-Resnick M. (2018). Brain Iron homeostasis: A focus on microglial Iron. Pharmaceuticals.

[22] Paul B.T., Manz D.H., Torti F.M., Torti S.V. (2017). Mitochondria and Iron: Current questions. Expert Rev. Hematol..

[23] Hamza I., Dailey H.A. (2012). One ring to rule them all: Trafficking of heme and heme synthesis intermediates in the metazoans. Biochim. Biophys. Acta Mol. Cell Res..

[24] Fujiwara T., Harigae H. (2015). Biology of heme in mammalian erythroid cells and related disorders. Biomed. Res. Int..

[25] Srour B., Gervason S., Monfort B., D’Autréaux B. (2020). Mechanism of iron–sulfur cluster assembly: In the intimacy of iron and sulfur encounter. Inorganics.

[26] Sukhbaatar N., Weichhart T. (2018). Iron regulation: Macrophages in control. Pharmaceuticals.

[27] Riemer J., Hoepken H.H., Czerwinska H., Robinson S.R., Dringen R. (2004). Colorimetric ferrozine-based assay for the quantitation of iron in cultured cells. Anal. Biochem..

[28] ImageJ. https://imagej.nih.gov/ij/.

[29] Bajpai V.K., Alam M.B., Quan K.T., Kwon K.R., Ju M.K., Choi H.J., Lee J.S., Yoon J.I., Majumder R., Rather I.A. (2017). Antioxidant efficacy and the upregulation of Nrf2-mediated HO-1 expression by (+)-lariciresinol, a lignan isolated from Rubia philippinensis, through the activation of p38. Sci. Rep..

[30] Scandalios J.G. (2005). Oxidative stress: Molecular perception and transduction of signals triggering antioxidant gene defenses. Braz. J. Med. Biol. Res..

[31] Forrester S.J., Kikuchi D.S., Hernandes M.S., Xu Q., Griendling K.K. (2018). Reactive oxygen species in metabolic and inflammatory signaling. Circ. Res..

[32] Simpson D.S.A., Oliver P.L. (2020). ROS generation in microglia: Understanding oxidative stress and inflammation in neurodegenerative disease. Antioxidants.

[33] Berlett B.S., Stadtman E.R. (1997). Protein oxidation in aging, disease, and oxidative stress. J. Biol. Chem..

[34] Jurga A.M., Paleczna M., Kuter K.Z. (2020). Overview of general and discriminating markers of differential microglia phenotypes. Front. Cell. Neurosci..

[35] Wang W.Y., Tan M.S., Yu J.T., Tan L. (2015). Role of pro-inflammatory cytokines released from microglia in Alzheimer’s disease. Ann. Transl. Med..

[36] Kim Y.S., Joh T.H. (2006). Microglia, major player in the brain inflammation: Their roles in the pathogenesis of Parkinson’s disease. Exp. Mol. Med..

[37] Kim S.R., Nakanishi K., Itagaki Y., Sparrow J.R. (2006). Photooxidation of A2-PE, a photoreceptor outer segment fluorophore, and protection by lutein and zeaxanthin. Exp. Eye Res..

[38] Xie K., Ngo S., Rong J., Sheppard A. (2019). Modulation of mitochondrial respiration underpins neuronal differentiation enhanced by lutein. Neural Regen. Res..

[39] Kabba J.A., Xu Y., Christian H., Ruan W., Chenai K., Xiang Y., Zhang L., Saavedra J.M., Pang T. (2018). Microglia: Housekeeper of the central nervous system. Cell. Mol. Neurobiol..

[40] Uttara B., Singh A., Zamboni P., Mahajan R. (2009). Oxidative stress and neurodegenerative diseases: A review of upstream and downstream antioxidant therapeutic options. Curr. Neuropharmacol..

[41] Chen X., Guo C., Kong J. (2012). Oxidative stress in neurodegenerative diseases. Neural Regen. Res..

[42] Lobo V., Patil A., Phatak A., Chandra N. (2010). Free radicals, antioxidants and functional foods: Impact on human health. Pharmacogn. Rev..

[43] Hasanuzzaman M., Bhuyan M.H.M.B., Zulfiqar F., Raza A., Mohsin S.M., Al Mahmud J., Fujita M., Fotopoulos V. (2020). Reactive oxygen species and antioxidant defense in plants under abiotic stress: Revisiting the crucial role of a universal defense regulator. Antioxidants.

[44] Giordano F.J. (2005). Oxygen, oxidative stress, hypoxia, and heart failure. J. Clin. Investig..

[45] Salim S. (2017). Oxidative stress and the central nervous system. J. Pharmacol. Exp. Ther..

[46] Ren X., Zou L., Zhang X., Branco V., Wang J., Carvalho C., Holmgren A., Lu J. (2017). Redox signaling mediated by thioredoxin and glutathione systems in the central nervous system. Antioxid. Redox Signal..

[47] Ransy C., Vaz C., Lombès A., Bouillaud F. (2020). Use of H2O2 to cause oxidative stress, the catalase issue. Int. J. Mol. Sci..

[48] Wang Y., Branicky R., Noë A., Hekimi S. (2018). Superoxide dismutases: Dual roles in controlling ROS damage and regulating ROS signaling. J. Cell Biol..

[49] Fukai T., Ushio-Fukai M. (2011). Superoxide dismutases: Role in redox signaling, vascular function, and diseases. Antioxid. Redox Signal..

[50] Mikhak B., Hunter D.J., Spiegelman D., Platz E.A., Wu K., Erdman J.W., Giovannucci E. (2008). Manganese superoxide dismutase (MnSOD) gene polymorphism, interactions with carotenoid levels and prostate cancer risk. Carcinogenesis.

[51] Ji G., Lv K., Chen H., Wang T., Wang Y., Zhao D., Qu L., Li Y. (2013). MiR-146a Regulates SOD2 Expression in H2O2 Stimulated PC12 Cells. PLoS ONE.

[52] Kaneko Y.S., Ota A., Nakashima A., Mori K., Nagatsu I., Nagatsu T. (2012). Regulation of oxidative stress in long-lived lipopolysaccharide-activated microglia. Clin. Exp. Pharmacol. Physiol..

[53] El Assar M., Angulo J., Rodríguez-Mañas L. (2013). Oxidative stress and vascular inflammation in aging. Free Radic. Biol. Med..

[54] Fresta C.G., Fidilio A., Lazzarino G., Musso N., Grasso M., Merlo S., Amorini A.M., Bucolo C., Tavazzi B., Lazzarino G. (2020). Modulation of pro-oxidant and pro-inflammatory activities of m1 macrophages by the natural dipeptide carnosine. Int. J. Mol. Sci..

[55] Kamoshita M., Toda E., Osada H., Narimatsu T., Kobayashi S., Tsubota K., Ozawa Y. (2016). Lutein acts via multiple antioxidant pathways in the photo-stressed retina. Sci. Rep..

[56] Mukherjee S., Forde R., Belton A., Duttaroy A. (2011). SOD2, the principal scavenger of mitochondrial superoxide, is dispensable for embryogenesis and imaginal tissue development but essential for adult survival. Fly (Austin).

[57] Lee K.H., Cha M., Lee B.H. (2020). Neuroprotective effect of antioxidants in the brain. Int. J. Mol. Sci..

[58] Fujimura M., Morita-Fujimura Y., Kawase M., Copin J.C., Calagui B., Epstein C.J., Chan P.H. (1999). Manganese superoxide dismutase mediates the early release of mitochondrial cytochrome C and subsequent DNA fragmentation after permanent focal cerebral ischemia in mice. J. Neurosci..

[59] Durante W. (2011). Protective role of heme oxygenase-1 against inflammation in atherosclerosis. Front. Biosci..

[60] Sun G.Y., Chen Z., Jasmer K.J., Chuang D.Y., Gu Z., Hannink M., Simonyi A. (2015). Quercetin attenuates inflammatory responses in BV-2 microglial cells: Role of MAPKs on the Nrf2 pathway and induction of heme oxygenase-1. PLoS ONE.

[61] Ko W., Yoon C.S., Kim K.W., Lee H., Kim N., Woo E.R., Kim Y.C., Kang D.G., Lee H.S., Oh H. (2020). Neuroprotective and anti-inflammatory effects of kuwanon c from cudrania tricuspidata are mediated by heme oxygenase-1 in ht22 hippocampal cells, raw264.7 macrophage, and bv2 microglia. Int. J. Mol. Sci..

[62] Matz P.G., Weinstein P.R., Sharp F.R. (1997). Heme oxygenase-1 and heat shock protein 70 induction in glia and neurons throughout rat brain after experimental intracerebral hemorrhage. Neurosurgery.

[63] Gozzelino R., Jeney V., Soares M.P. (2010). Mechanisms of cell protection by heme oxygenase-1. Annu. Rev. Pharmacol. Toxicol..

[64] Parfenova H., Leffler C.W., Basuroy S., Liu J., Fedinec A.L. (2012). Antioxidant roles of heme oxygenase, carbon monoxide, and bilirubin in cerebral circulation during seizures. J. Cereb. Blood Flow Metab..

[65] Korolnek T., Hamza I. (2014). Like iron in the blood of the people: The requirement for heme trafficking in iron metabolism. Front. Pharmacol..

[66] Donegan R.K., Moore C.M., Hanna D.A., Reddi A.R. (2019). Handling heme: The mechanisms underlying the movement of heme within and between cells. Free Radic. Biol. Med..

[67] Ndayisaba A., Kaindlstorfer C., Wenning G.K. (2019). Iron in neurodegeneration—Cause or consequence?. Front. Neurosci..

[68] Paradkar P.N., Zumbrennen K.B., Paw B.H., Ward D.M., Kaplan J. (2009). Regulation of mitochondrial iron import through differential turnover of mitoferrin 1 and mitoferrin 2. Mol. Cell. Biol..

[69] Li F.Y., Nikali K., Gregan J., Leibiger I., Leibiger B., Schweyen R., Larsson C., Suomalainen A. (2001). Characterization of a novel human putative mitochondrial transporter homologous to the yeast mitochondrial RNA splicing proteins 3 and 4. FEBS Lett..

[70] Hunter G.A., Al-Karadaghi S., Ferreira G.C. (2011). Ferrochelatase: The convergence of the porphyrin biosynthesis and iron transport pathways. J. Porphyr. Phthalocyanines.

[71] Sachar M., Anderson K.E., Ma X. (2016). Protoporphyrin IX: The good, the bad, and the ugly. J. Pharmacol. Exp. Ther..

[72] Santambrogio P., Biasiotto G., Sanvito F., Olivieri S., Arosio P., Levi S. (2007). Mitochondrial ferritin expression in adult mouse tissues. J. Histochem. Cytochem..

[73] Yang H., Yang M., Guan H., Liu Z., Zhao S., Takeuchi S., Yanagisawa D., Tooyama I. (2013). Mitochondrial ferritin in neurodegenerative diseases. Neurosci. Res..

[74] Rouault T.A. (2016). Mitochondrial iron overload: Causes and consequences. Curr. Opin. Genet. Dev..

[75] Tong W.H., Rouault T.A. (2006). Functions of mitochondrial ISCU and cytosolic ISCU in mammalian iron-sulfur cluster biogenesis and iron homeostasis. Cell Metab..

[76] Lill R., Dutkiewicz R., Elsässer H.P., Hausmann A., Netz D.J.A., Pierik A.J., Stehling O., Urzica E., Mühlenhoff U. (2006). Mechanisms of iron-sulfur protein maturation in mitochondria, cytosol and nucleus of eukaryotes. Biochim. Biophys. Acta Mol. Cell Res..

[77] Linton K.J., Higgins C.F. (2007). Structure and function of ABC transporters: The ATP switch provides flexible control. Pflug. Arch. Eur. J. Physiol..

[78] Richardson D.R., Lane D.J.R., Becker E.M., Huang M.L.H., Whitnall M., Rahmanto Y.S., Sheftel A.D., Ponka P. (2010). Mitochondrial iron trafficking and the integration of iron metabolism between the mitochondrion and cytosol. Proc. Natl. Acad. Sci. USA.

[79] Bordt E.A., Polster B.M. (2014). NADPH oxidase- and mitochondria-derived reactive oxygen species in proinflammatory microglial activation: A Bipartisan affair?. Free Radic. Biol. Med..

[80] Wu W., Li Y., Wu Y., Zhang Y., Wang Z., Liu X. (2015). Lutein suppresses inflammatory responses through Nrf2 activation and NF-κB inactivation in lipopolysaccharide-stimulated BV-2 microglia. Mol. Nutr. Food Res..

[81] Kim J.E., Clark R.M., Park Y., Lee J., Fernandez M.L. (2012). Lutein decreases oxidative stress and inflammation in liver and eyes of guinea pigs fed a hypercholesterolemic diet. Nutr. Res. Pract..

[82] Ip W.K.E., Hoshi N., Shouval D.S., Snapper S., Medzhitov R. (2017). Anti-inflammatory effect of IL-10 mediated by metabolic reprogramming of macrophages. Science (80-).

[83] Meng J., Ni J., Wu Z., Jiang M., Zhu A., Qing H., Nakanishi H. (2018). The critical role of IL-10 in the antineuroinflammatory and antioxidative effects of Rheum tanguticum on activated microglia. Oxid. Med. Cell. Longev..

[84] DePaula-Silva A.B., Gorbea C., Doty D.J., Libbey J.E., Sanchez J.M.S., Hanak T.J., Cazalla D., Fujinami R.S. (2019). Differential transcriptional profiles identify microglial- and macrophage-specific gene markers expressed during virus-induced neuroinflammation. J. Neuroinflamm..

[85] Giustarini D., Dalle-Donne I., Tsikas D., Rossi R. (2009). Oxidative stress and human diseases: Origin, link, measurement, mechanisms, and biomarkers. Crit. Rev. Clin. Lab. Sci..

[86] Wang M., Tsao R., Zhang S., Dong Z., Yang R., Gong J., Pei Y. (2006). Antioxidant activity, mutagenicity/anti-mutagenicity, and clastogenicity/anti-clastogenicity of lutein from marigold flowers. Food Chem. Toxicol..

