# Supplementary material for: Lutein Exerts Antioxidant and Anti-Inflammatory Effects and Influences Iron Utilization of BV-2 Microglia
Source: Antioxidants (Basel). 2021 Feb 27;10(3):363. doi: 10.3390/antiox10030363 (PMC7997267; doi:10.3390/antiox10030363)
Supplement: Supplementary file 1 [file antioxidants-10-00363-s001.zip › supplementary/Supplementary materials figure legends.docx]

Supplementary materials figure legends

**Figure S1**. Cell viability determinations of the lutein treated BV-2 cells. Cell viability measurements of lutein and DMSO treated BV-2 cells. Cell viability assays were made in quadruplicate in three independent experiments. The bars represent mean values and error bars represent standard deviation (SD) for three independent experiments (n=3). The * indicates p < 0.05 compared to the appropriate DMSO control at 6 h, 24 h or 48 h. Abbreviations of treatments: Control-absolute control; DMSO controls: D7-DMSO equivalent to 7.5 ng/µL of lutein; D10-DMSO equivalent to 10 ng/µL of lutein; D5- DMSO equivalent to 5 ng/µL of lutein; D2-DMSO equivalent to 2.5 ng/µL of lutein.

**Figure S2**. Cell viability determinations of the H_2_O_2_ treated BV-2 cells. Cell viability measurements of H_2_O_2_ treated BV-2 cells. Cell viability assays were made in quadruplicate in three independent experiments. The bars represent mean values and error bars represent standard deviation (SD) for three independent experiments (n=3). The † indicates p < 0.05 compared to the control at 2 h, 6 h, 24 h or 48 h. Abbreviations of treatment: Control-absolute control.

**Table S1.** Cell viability determinations of the lutein with H_2_O_2_ treated BV-2 cells. Cell viability assays were made in quadruplicate in three independent experiments. The bars represent mean values of percentage of living cells and ± represents standard deviation (SD) for three independent experiments (n=3). Abbreviations of treatments: Control-absolute control; DMSO controls: D7-DMSO equivalent to 7.5 ng/µL of lutein; D10-DMSO equivalent to 10 ng/µL of lutein.
