# Supplementary material for: Lutein Exerts Antioxidant and Anti-Inflammatory Effects and Influences Iron Utilization of BV-2 Microglia
Source: Antioxidants (Basel). 2021 Feb 27;10(3):363. doi: 10.3390/antiox10030363 (PMC7997267; doi:10.3390/antiox10030363)
Supplement: Supplementary file 1 [file antioxidants-10-00363-s001.zip › supplementary/Table S1.docx]

**Table S1.** Cell viability determinations of the lutein with H_2_O_2_ treated BV-2 cells. Cell viability assays were made in quadruplicate in three independent experiments. The bars represent mean values of percentage of living cells and ± represents standard deviation (SD) for three independent experiments (n=3). Abbreviations of treatments: Control-absolute control; DMSO controls: D7-DMSO equivalent to 7.5 ng/µL of lutein; D10-DMSO equivalent to 10 ng/µL of lutein.

| **Time** | **Lutein** | | **H_2_O_2_** | **Lutein + H_2_O_2_** | | **H_2_O_2_** | **Lutein + H_2_O_2_** | | **Control** | **DMSO Control** | |
| --- | --- | --- | --- | --- | --- | --- | --- | --- | --- | --- | --- |
|  | **7.5 ng/µL** | **10 ng/µL** | **5 µM** | **5 µM + 7.5 ng/µL** | **5 µM + 10 ng/µL** | **10 µM** | **10 µM + 7.5 ng/µL** | **10 µM + 10 ng/µL** |  | **D7** | **D10** |
| 6 h | 110,7 ± 1,25 | 113,41 ± 2,88 | 95,33 ± 1,17 | 96,15 ± 1,31 | 99,48 ± 0,24 | 93,08 ± 2,41 | 94,62 ± 0,99 | 96,65 ± 1,09 | 100 | 96,38 ± 2,42 | 95,46 ± 1,55 |
| 24 h | 125,29 ± 2,38 | 132,8 ± 4,32 | 95,12 ± 1,41 | 95,89 ± 2,19 | 97,46 ± 1,49 | 89,99 ± 5,2 | 93,47 ± 2,49 | 95,11 ± 1,86 | 100 | 94,21 ± 1,64 | 89,99 ± 1,31 |
| 48 h | 148,03 ± 3,11 | 153,09 ± 2,91 | 89,92 ± 3,07 | 92,3 ± 1,15 | 92,67 ± 1,78 | 85,99 ± 4,04 | 92,15 ± 1,86 | 94,19 ± 3,23 | 100 | 91,27 ± 1,1 | 86,93 ± 1,43 |
