# Supplementary figures and images for: Lutein Exerts Antioxidant and Anti-Inflammatory Effects and Influences Iron Utilization of BV-2 Microglia
Source: Antioxidants (Basel). 2021 Feb 27;10(3):363. doi: 10.3390/antiox10030363 (PMC7997267; doi:10.3390/antiox10030363)

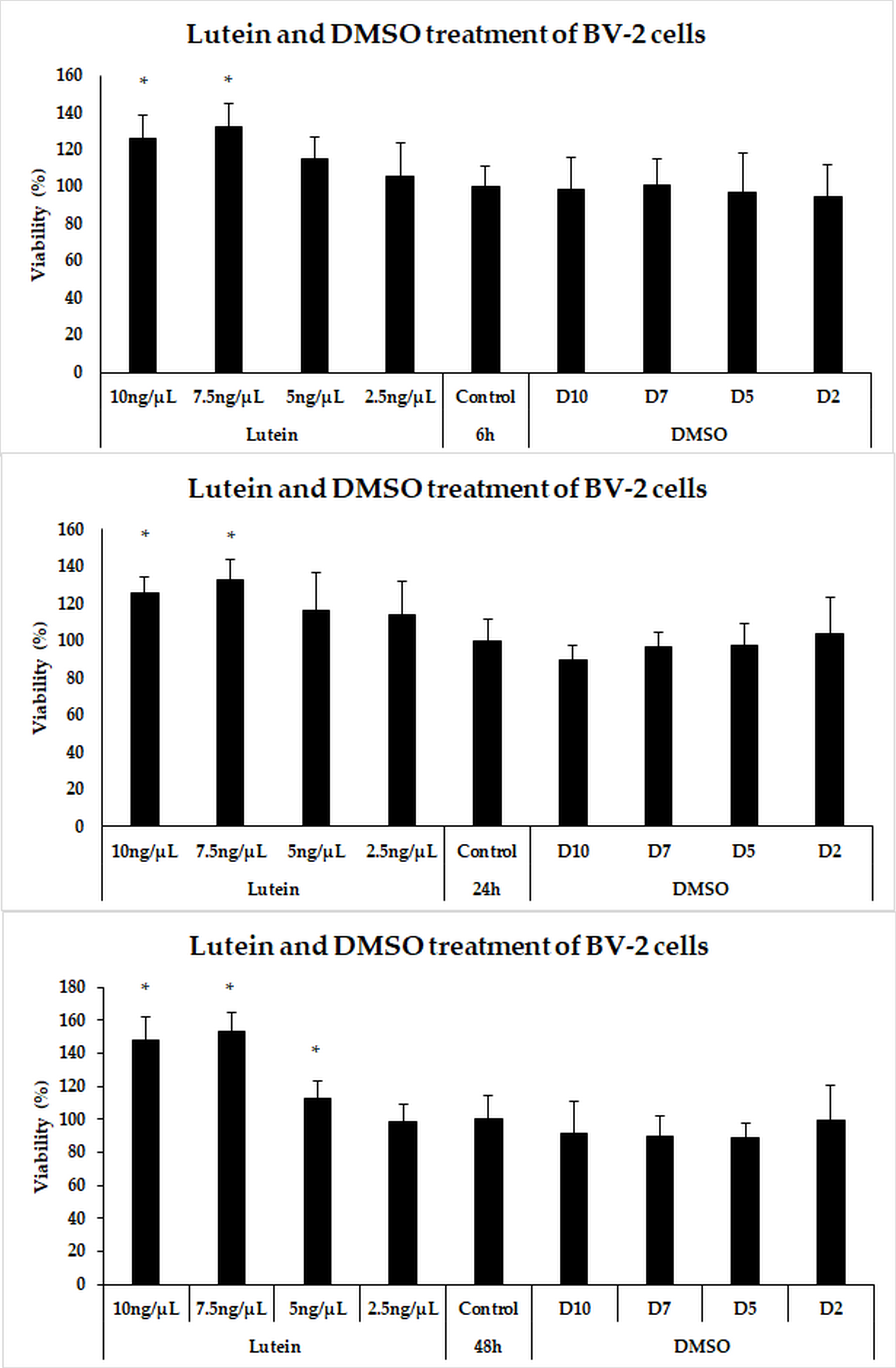

Supplement: Supplementary file 1 [file antioxidants-10-00363-s001.zip › supplementary/Figure S1.tiff]

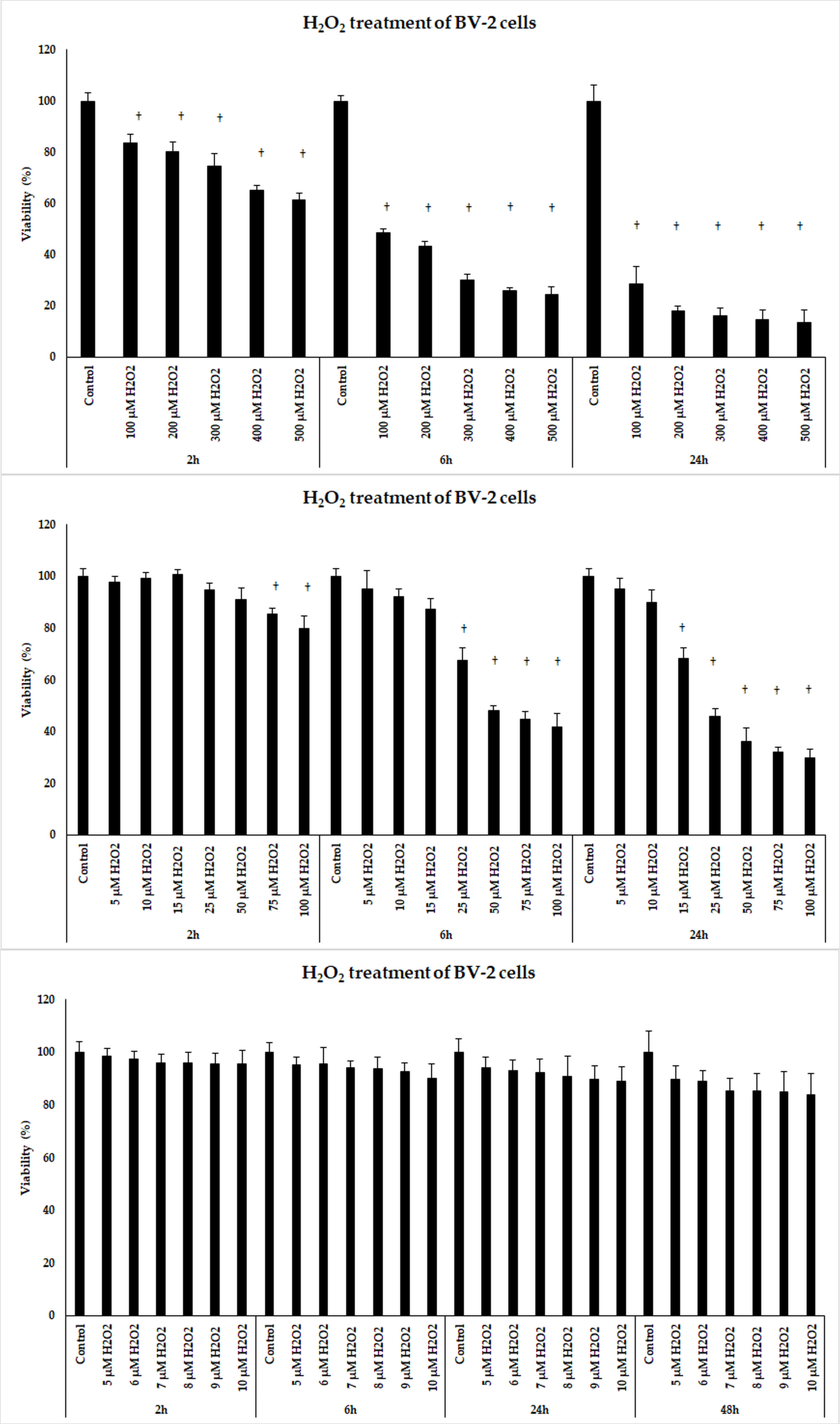

Supplement: Supplementary file 1 [file antioxidants-10-00363-s001.zip › supplementary/Figure S2.tiff]
